# Unlocking the microbiome of an extremophile plant: metagenomic insights into *Calotropis procera*’s endo-rhizosphere communities

**DOI:** 10.1007/s11274-026-04902-4

**Published:** 2026-03-24

**Authors:** Thamara de Medeiros Azevedo, Flávia Figueira Aburjaile, Valesca Pandolfi, José Ribamar Costa Ferreira-Neto, Giselle Gomes Monteiro Fracetto, Roberta Lane de Oliveira Silva, Rodrigo César Gonçalves-Oliveira, Vasco Ariston de Carvalho Azevedo, Bertram Brenig, Ana Maria Benko-Iseppon

**Affiliations:** 1 Departamento de Genética, Centro de Biociências, Universidade Federal de Pernambuco, Av. Prof. Moraes Rego, Cidade Universitária, Recife, PE/CEP: 50670-901 Brazil; 2 Departamento de Medicina Veterinária Preventiva, Universidade Federal de Minas Gerais, Belo Horizonte, MG Brazil; 3 Embrapa Soja – Brazilian Agricultural Research Corporation (Embrapa), Distrito de Warta, Londrina, PR Brazil; 4 Laboratório de Microbiologia E Bioquímica Do Solo, Universidade Federal Rural de Pernambuco, Recife, PE Brazil; 5 Universidade de Pernambuco, Petrolina, PE Brazil; 6 Departamento de Genética, Universidade Federal de Minas Gerais, Ecologia E Evolução, Belo Horizonte, MG Brazil; 7 Department of Molecular Biology of Livestock, University Göttingen, Göttingen, Germany

**Keywords:** Caatinga, Coastal sandy soil, OTUs, Restinga, Semi-arid clayey soil

## Abstract

This study explores the root-associated microbiome of *Calotropis procera*, a drought-adapted, invasive plant thriving in Brazil. We analyzed microbial communities from the root endosphere, rhizosphere, and adjacent soil in two contrasting ecosystems: Caatinga (semi-arid) and Restinga (coastal). Using 16S rDNA sequencing and shotgun metagenomics, we tested three hypotheses: (I) environmental specificity of the rhizospheric bacterial microbiome, (II) continuity of bacterial composition between bulk soil and rhizosphere, and (III) host-driven filtering of the endophytic microbiome. Despite differing soil conditions – more sodium in Restinga and higher organic carbon in Caatinga – microbial profiles in root compartments remained consistent. The root endosphere was enriched with stress-tolerant bacteria and novel archaea, while fungal genera included *Fusarium* and *Puccinia*. Results partially supported environmental specificity and showed moderate soil-rhizosphere continuity, with evidence of plant-mediated selection. Host filtering was evident for bacteria and fungi but not archaea. These data indicate a *C. procera*-mediated regulation of its root microbiome composition, whereby the plant may either selectively recruit specific taxa from prevalent soil microbial communities (e.g., through root exudates) or vertically transmit a conserved subset of its microbiome via seeds. Our study enhances understanding of the *C. procera* microbiome and its microbial interactions, identifying potential candidates for future biotechnological applications.

**Supplementary Information:**

The online version contains supplementary material available at 10.1007/s11274-026-04902-4.

## Introduction

Plants harbor a dynamic consortium of microorganisms, collectively termed the plant microbiome (Trivedi et al. 2020). This entity performs a series of interactions that span a spectrum of ecological outcomes – mutualistic, commensal, or pathogenic – each differentially impacting host fitness (Hassani et al. 2019; Acuña and Jorquera 2020; de Meiros Azevedo et al. 2021).

In the positive context, the microbiome can play essential roles for the host plant, such as improving nutrient acquisition, growth promotion, and tolerance to (a)biotic stresses (Dastogeer et al. 2020). This cooperative interplay between plants and their inhabitants, holistically conceptualized as a holobiont organism, exemplifies a co-evolved adaptive strategy that optimizes host survival and ecological competitiveness (Zilber-Rosenberg and Rosenberg 2008).

The plant’s microbial colonization occurs generically in two distinct niches: episphere (on plant surfaces) and endosphere (within internal tissues). The rhizosphere, an episphere element defined as the narrow soil zone surrounding plant roots, plays a pivotal role in plant health and ecosystem functioning by mediating nutrient cycling, microbial interactions, and stress resilience. The composition of that microbiome is modulated by soil type, host genotype, developmental phenology, and by root exudates, which act as chemical filters that selectively attract or inhibit microbial taxa (Chaparro et al. 2014; Qu et al. 2020; de Meiros Azevedo et al. 2021). This region is characterized by high microbial density, driven by plant exudation of primary and secondary metabolites, which not only attract beneficial microbial consortia but also serve as chemical deterrents against pathogenic invaders (Venturi and Keel 2016).

The endosphere, in turn, hosts a specialized community of endophytic microorganisms that play critical roles in plant growth, stress adaptation, and disease resistance. Such “guests” are so vital to plant health that they can be vertically transmitted across generations. In seed-bearing plants, seeds harbor a relevant microbiome and serve as crucial vectors, bridging successive generations and ensuring the continuity of beneficial microbial partnerships (Abdelfattah et al. 2021, 2023). Endophytes colonize roots, stems, leaves, and seeds without causing harm, forming mutualistic relationships that enhance nutrient acquisition, such as nitrogen fixation and phosphate solubilization (Compant et al. 2019; de Meiros Azevedo et al. 2021). The successful penetration and colonization of these organisms depend on plant features (e.g., genotype and tissue type), microorganisms themselves (e.g., colonizing species and their dissemination mechanisms), besides biotic and abiotic factors (Krishnaraj and Pasha 2017). Consequently, the root endosphere often exhibits lower microbial diversity compared to the surrounding soil microbial communities (e.g., rhizosphere) (Bulgarelli et al. 2012; Edwards et al. 2015). The endophytic niche offers protection for the microbial community and concomitantly enables intimate contact of endophytes with plant tissues, directly supplying beneficial effects (Ryan et al. 2008; Afzal et al. 2019).

Given the wealth of information presented above and the beneficial impact of some microorganisms on plant molecular physiology, analyzing microbiomes associated with extremophilic flora emerges as a highly promising strategy (Backer et al. 2018; Ajar Nath 2021). In this context, the Brazilian northeastern semi-arid region constitutes a biodiversity hotspot of unparalleled scientific interest. Encompassing approximately 18% of the nation’s territory, this region is dominated by semi-arid ecosystems, with the Caatinga biome – a flagship of South America’s Seasonally Dry Tropical Forests – serving as its ecological cornerstone (Pinheiro et al. 2013). The Caatinga is distinguished by extraordinary floristic diversity, harboring a multitude of species adapted to poor soils, chronic water deficit and extreme climatic variability (Franca-Rocha et al. 2007; de Sá et al. 2021). This evolutionary resilience positions the Caatinga’s plants and respective microbiomes as an underexplored treasure trove for discovering stress-adaptive microbial symbionts, offering transformative potential for agricultural innovation in arid regions.

A species of particular ecological interest within the Caatinga biome is *Calotropis procera* (Apocynaceae), an evergreen shrub indigenous to Africa and Asia (Grace 2006). Renowned for its exceptional abiotic stress tolerance – notably to drought and salinity – *C. procera* has proliferated across arid and semi-arid regions globally (Hassan et al. 2015; Muhammed et al. 2017; Hassan 2017). Introduced to Brazil, *C. procera* has become an invasive exotic species spreading across several regions of South America, mainly in dry biomes, where it occurs in contrasting ecosystems such as Caatinga, Restinga (a coastal ecosystem characterized by sandy and saline terrain), Cerrado (fire-adapted savannas), and degraded urban environments (Silva et al. 2010; Sobrinho et al. 2013; Nascimento et al. 2015; Gonçalves-Oliveira et al. 2022). Few plant species exhibit such broad adaptive capacity across disparate environmental gradients. The ability of *C. procera* to thrive in these ecologically divergent habitats underscores its extraordinary phenotypic and physiological versatility, positioning it as a compelling candidate for investigating plant-microbiome synergies. However, the structural and functional dynamics of its associated microbiome remain an underexplored frontier, particularly in relation to its colonization of contrasting ecosystems.

Given the context as mentioned above, this study aimed to characterize the structural and compositional diversity of the root endophytic, rhizosphere, and adjacent soil microbiomes of *C. procera* across two highly divergent ecosystems: the Caatinga (a seasonally dry tropical forest unique to Brazil) and Restinga (coastal sand plains). To elucidate the interaction between environmental gradients, host selection, and microbial assembly, the following hypotheses were tested:

I. Environmental specificity hypothesis: Contrasting ecosystems (Caatinga and Restinga) are expected to harbor distinct rhizosphere bacterial microbiome in *C. procera*, shaped by local environmental conditions.
II. Soil-rhizosphere continuity hypothesis: Rhizosphere bacterial composition correlates significantly with adjacent soil communities, reflecting environmental imprinting;
III. Host filtering hypothesis: The root endosphere microbiome is predominantly shaped by host-specific selection, transcending environmental variability.

To scrutinize these points, we employed a dual metagenomic strategy (shotgun metagenomic and 16S rDNA amplicon sequencing) to identify the predominant microbial taxa composing the rhizosphere, adjacent soil, and root endosphere communities of *C. procera*, as well as to compare the endosphere structure and diversity across both environments.

## Materials and methods

### Sample collection

**Fig. 1:**
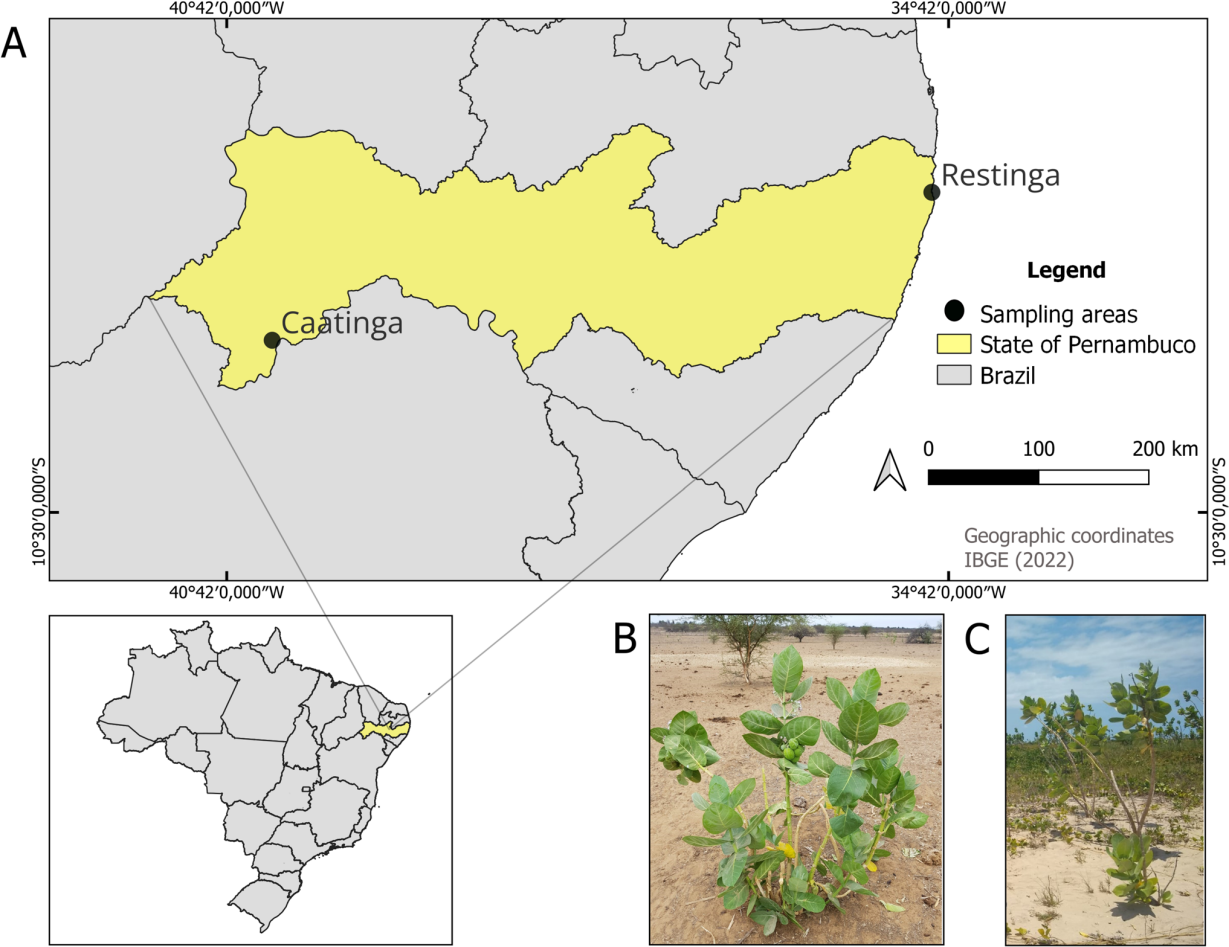
Geographical location of sampling points in Pernambuco, Brazil **A**. Images of *C. procera* in the Caatinga **B** and Restinga **C** environments

Triplicate biological replicates of *C. procera* root systems (with adhering rhizospheric soil) and adjacent bulk soil were obtained from two georeferenced populations in contrasting Brazilian environments (Fig. 1A):

- Restinga Environment (Fig. 1B): Coastal sand plains at 7°50′26.9"S, 34°50′25.0"W (Paulista municipality, Pernambuco state);
- Caatinga Environment (Fig. 1C: Seasonally dry tropical forest at 9°04′15.6"S, 40°19′21.5"W (Petrolina municipality, Pernambuco state).

The collecting points were 640.4 km apart (Fig. 1A) and significantly differed in soil composition, climate, and rainy season. Samples were collected during the rainy season in both areas, i.e., in September 2019 (Restinga) and February 2020 (Caatinga). For each location, three plants with approximately the same height (approximately 1.50 m) and reproductive developmental stage were selected in a homogeneous area, considering a distance of at least 5 m from each other. The selected plants were carefully excavated using a shovel.

The plants were gently shaken to remove excess soil. Rhizospheric soil (i.e., soil adhering to and released from the roots) was collected from each plant using sterilized brushes and transferred into sterile 50 mL tubes. Multiple root segments from each plant were then excised and placed in separate sterile 50 mL tubes. Both sample types (rhizosphere and roots) were stored on ice and transported to the laboratory. After surface-sterilized (immersion in 70% alcohol for 1 min, sodium hypochlorite solution (2.5%) for 3 min, and alcohol 70% for 30 s, followed by washing three times in sterile distilled water (Araújo et al. 2001), the roots were stored at −80ºC until DNA extraction.

Soil samples (0–20 cm depth) adjacent to the plants were also collected in triplicate, constituting two subsamples: a subsample (collected into sterile 50 ml tubes) for molecular analysis and another subsample (placed in plastic bags) sent to Soil Environmental Chemistry Laboratory (Federal Rural University of Pernambuco—UFRPE, Recife, PE, Brazil) for chemical characterization (Figure S1).

### Soil chemical analysis

Chemical analyses of soil were performed as described by Teixeira et al. (2017). Initially, the soil samples were air-dried, homogenized, and passed through a 2 mm sieve, obtaining air-dried fine soil. Subsequently, soil pH was measured using a water solution 1: 2.5 (soil: solution). The exchangeable K^+^ and Na^+^ were extracted with Mehlich^−1^ solution (HCl 0,05 mol L^−1^ and H_2_SO_4_ 0,0125 mol L^−1^) and measured by flame photometry. Available phosphorus was extracted with Mehlich^−1^ and determined by colorimetric analysis. Exchangeable Ca_2_^+^ and Mg_2_^+^ were determined by the volumetric method by titration with EDTA after extraction with potassium chloride (KCl) solution 1 mol L^−1^, exchangeable Al^3+^ was determined by the volumetric method by titration with sodium hydroxide, and after extraction with KCl 1 mol L^−1^. H + Al was determined from extraction with 0.5 mol L^−1^ calcium acetate solution, followed by alkalimetric titration. Organic Carbon (OC) and Organic Matter (OM) were determined by the Walkley–Black method (Walkley and Black 1934). To analyze the differences in the chemical parameters of the soils adjacent to the plants in Restinga and Caatinga environments, the t-test (*p* < 0.05) was used, with the aid of the R software version 4.2.2.

### DNA extraction, amplification, and sequencing of 16S rDNA

Total genomic DNA was isolated from the rhizosphere and adjacent soil regions employing the DNeasy PowerSoil kit (QIAGEN, Germany). For *C. procera* root samples, superficial sterilization was conducted prior to cryogenic maceration in liquid nitrogen, followed by pulverization into a homogenized powder using sterile mortars and pestles. Subsequent DNA extraction was executed with the DNeasy PowerPlant Pro kit (QIAGEN), adhering strictly to the manufacturer’s protocol. DNA purity and concentration were assessed via a NanoDrop 2000c spectrophotometer (Thermo Fisher Scientific, USA), while structural integrity was verified through 1% agarose gel electrophoresis. Quantitative precision was ensured using a Qubit 2.0 fluorimeter (Thermo Fisher Scientific) in conjunction with the Qubit™ dsDNA HS Assay Kit (Invitrogen, USA).

For library construction, DNA aliquots were processed with the TruSeq Library Prep Kit (Illumina, USA), targeting the hypervariable V3/V4 regions of the 16S rDNA through amplification with the 341F/806R primer pair (Caporaso et al. 2010). Sequencing pools were normalized to a concentration of 17.5 pM and subjected to high-throughput sequencing on the MiSeq platform (Illumina) utilizing the V3 × 600 sequencing kit. Paired-end runs (2 × 500 cycles) were conducted, achieving an average sequencing depth of 100 × coverage per sample.

### Bioinformatic processing and statistical evaluation of 16S rRNA amplicon sequencing data for bacterial microbiome diversity analysis

Trimmomatic (Bolger et al. 2014) was used for quality filtering of the obtained FASTQ files, removing truncated and low-quality reads (phred score < 20). Subsequently, forward and reverse paired reads were merged, and the sequences were subjected to dereplication, abundance classification, singleton removal, and chimera filtering with Mothur software (Schloss et al. 2009). After that, pre-processed sequences were grouped into Taxonomic Operational Units (OTU) (≥ 97% similarity) and assigned taxonomically using QIIME (Caporaso et al. 2010) based on a sequence similarity threshold of 97% against the database of SILVA (https://www.arbsilva.de/).

Statistical analyses of amplicon sequencing data were performed within the R (v. 4.2.2) statistical environment. The relative abundance of bacterial taxa was displayed in percentage bar graphs. Subsequently, the OTUs tables were normalized through sizing with Scaling with Ranked Subsampling with the help of the “SRS” function in the “SRS” package (Beule and Karlovsky 2020). Based on the normalized tables, the alpha diversity of the bacterial community was estimated using three metrics: (1) Chao1 index (community richness estimator based on abundance); (2) Shannon index (considers both species richness and evenness); and (3) Pielou's equitability (represents the evenness of the community), through the “estimate_richness” and “diversity” functions of the "phyloseq" (McMurdie and Holmes 2013) and "vegan" packages (Oksanen et al. 2022). Kruskal–Wallis tests were used to compare the results obtained for alpha diversity indices. In the case of comparisons in which the test was significant, post hoc Dunn tests corrected by Bonferroni were performed. Box plots were created to visualize alpha diversity indices using the “ggplot2” package (Wickham 2009).

Differences in bacterial microbiome composition between niches and environments were estimated using the Bray–Curtis dissimilarity matrix, which was later used to create the Principal Coordinate Analysis (PCoA) graph in the “ggplot2” package. The effect of niche type (rhizosphere x adjacent soil) and sampled region (Restinga x Caatinga), as well as the interaction between both on the composition of the bacterial community, was determined using Multivariate Permutation Analyses of Variance (PERMANOVA) with 999 permutations by executing the “adonis2” function of “vegan”. In addition, the permutational multivariate dispersion analysis was performed using the “betadisper” function to test the homogeneity of the multivariate dispersion within the groups, followed by the permutation test using the “permutest” function (“vegan” package), with FDR-adjusted p-values.

Redundancy analysis (RDA) was performed on the “vegan” package to determine the correlation between bacterial community structure and soil chemical properties. First, the matrices were analyzed using Detrended Correspondence Analysis (DCA) to estimate the size of the gradient of the species distribution, resulting in linearly distributed data (gradient length < 3), indicating RDA as the most appropriate model for the data. To verify the significance of the chemical properties of the soil on the bacterial community, direct selection (FS) and Monte Carlo permutation tests were performed with 1000 random permutations. Venn diagrams were also plotted with the “venn.diagram” function of the "VennDiagram" package (Chen and Boutros 2011) to visualize the number of shared and exclusive OTUs between groups of soil and rhizosphere samples.

To determine the pattern of interactions between bacterial communities in each niche and environment, network analysis between bacterial genera from the rhizosphere and adjacent soils was calculated from sparse correlations for compositional data (SparCC). In the analysis of the SparCC (Friedman and Alm 2012), OTUs with relative abundance greater than 0.01% were maintained, and correlations with coefficients > 0.5 or < −0.5 and *p*-values < 0.05 were considered robust and included for the generation of networks. Each node in the reconstructed networks represents a genus, and each edge corresponds to significant correlations (positive or negative) between two nodes. The number of nodes, edges, network diameter (greatest distance between nodes), and average degree (average number of edges connected to each node) were calculated, and the co-occurrence networks were visualized using the Cytoscape software (version 3.9.1) (Shannon et al. 2003).

### Shotgun library construction, metagenomic sequencing, and data processing

Shotgun metagenomic libraries were generated from *C. procera* root-derived DNA using the Nextera XT Library Preparation Kit (Illumina, USA). Following enzymatic fragmentation, purified DNA fragments underwent adapter ligation and library quantification via qPCR, after which paired-end sequencing (2 × 150 bp) was conducted on the Illumina HiSeq 2500 platform under stringent quality thresholds. Raw sequencing data underwent initial quality assessment with FastQC (v.0.12.1; Babraham Bioinformatics), followed by adapter trimming and read filtration using Trimmomatic (v.0.32) with the following parameters: ILLUMINACLIP:TruSeq3-PE-2.fa:2:30:10, SLIDINGWINDOW:4:30, and MINLEN:50 to retain only high-fidelity reads (Phred score ≥ 30).

Taxonomic profiling of Bacteria, Fungi, and Archaea was accomplished via the Lowest Common Ancestor (LCA) algorithm, leveraging annotated reference sequences from the SILVA (v.138.1) (https://www.arb-silva.de/), Greengenes (v.13_8) (https://greengenes.secondgenome.com/), and Ribosomal Database Project (RDP, v.11.5) (http://rdp.cme.msu.edu/) databases.

### Statistical analyses of shotgun data

The relative abundances of major microbial communities at phyla and genera levels were visualized in GraphPad Prism version 8.0.2 (http://www.graphpad.com). Data analysis was conducted in R (v. 4.2.2). The alpha taxonomic diversity estimate was calculated based on the Chao1, Shannon, and Evenness indices as described above. The diversity indexes were compared between root endosphere samples of *C. procera* from both collected regions using the t-test. PCoA was performed to assess the beta diversity based on the Bray–Curtis distance between the taxonomic profile of microbial communities of the root endosphere in the environments studied. Bray–Curtis distances were used as input to PERMANOVA, using the “adonis” function in the “vegan” R package with 999 permutations, to assess differences in the composition of microbial communities between sample groups.

### Declaration of artificial inteligence use

This manuscript includes content that was translated and revised with the assistance of an artificial intelligence tool (ChatGPT, OpenAI). The AI tool was used exclusively to support language translation and text revision. All scientific content, analysis, interpretation, and conclusions are the sole responsibility of the authors.

## Results

### Comparative analysis of soil chemical properties in Caatinga and Restinga ecosystems

The chemical characteristics of the adjacent soil to the *C. procera* plants from both studied areas are presented in Table 1. The Caatinga and Restinga soils were characterized as acidic (Table 1), although the non-detection by the Al^3+^ analysis and the “H + Al” contents significantly lower than those presented by the Caatinga soil may have contributed to the higher pH values of the Restinga soil samples (Table 1). The available phosphorus (P) content was significantly higher in the Restinga soil (11.64 ± 0.14 mg dm^−3^) compared to Caatinga (2.25 ± 0.9153 mg dm^−3^) (Table 1).

**Table 1:** Chemical properties of soils in the analyzed environments

Parameters	Soil samples	
	Restinga	Caatinga
*pH	6.30 ± 0.26	5.03 ± 0.20
Ca^2+^	1.46 ± 0.05	1.63 ± 0.15
Mg^2+^	0.066 ± 0.11	0.13 ± 0.057
Al^3+^	0.0 ± 0.0	0.083 ± 0.057
*Na^+^	0.48 ± 0.032	0.22 ± 0.037
K^+^	0.020 ± 0.0	0.28 ± 0.10
*P	11.64 ± 0.14	2.25 ± 0.9153
*OC	0.95 ± 0.17	3.50 ± 0.091
*OM	1.65 ± 0.29	6.04 ± 0.15
*H + Al	0.58 ± 0.095	2.46 ± 0.35

^*^Significant differences between Restinga vs Caatinga were detected using the t-test (*p* < 0.05)

On the other hand, the organic carbon (OC) and organic matter (OM) contents were significantly higher in the Caatinga soil samples. As for the concentration of the other analyzed macro and micronutrients (Ca^2+^, Mg^2+^ and K^+^), there was no significant difference between both soil types. The exception was Na^+^, whose content in Restinga soil was significantly higher (Table 1).

Number of biological replicates (*n*) = 3. Data are presented as mean ± standard deviation. Units: available phosphorus (P) in mg dm^−3^; exchangeable calcium (Ca^2+^), exchangeable magnesium (Mg^2+^), aluminum (Al^3+^), exchangeable acidity, exchangeable sodium (Na^+^), exchangeable potassium (K^+^), and potential acidity in cmolcdm^−3^; organic carbon (OC) and organic matter (OM) in g kg^−1^. *Significant differences between Restinga and Caatinga soils were detected using the t-test (*p* < 0.05).

### Soil chemical properties vs. bacterial community

**Fig. 2:**
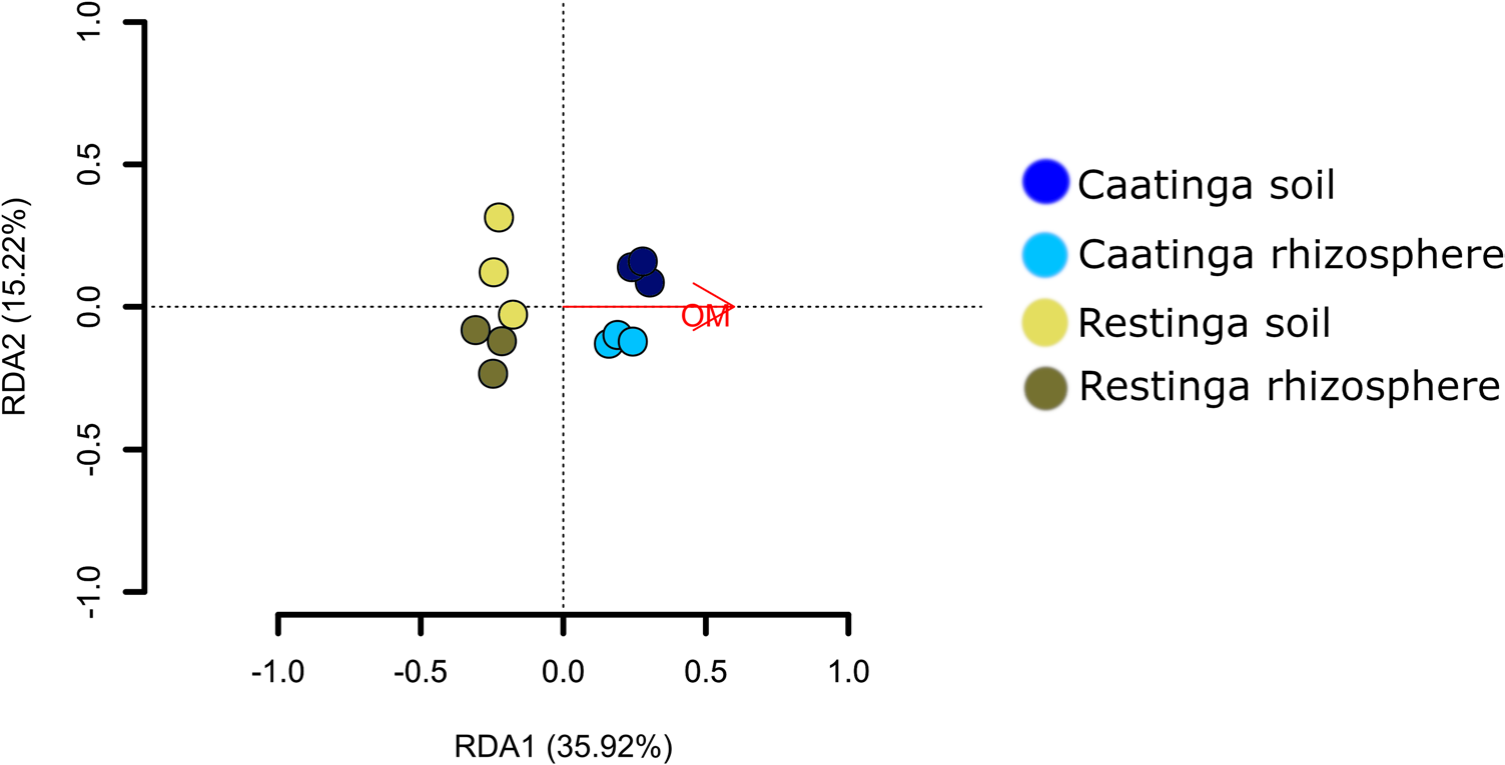
Redundancy analysis (RDA) of bacterial community composition at the genus level in relation to soil chemical properties. Only the variable organic matter (OM), which showed a significant association (*P* < 0.05), is shown

The relationship between the bacterial taxonomic composition of adjacent soil and rhizosphere samples with environmental variables (soil properties) was assessed using Redundancy Analysis (RDA), with significance tested via Monte Carlo permutation. The first two RDA axes collectively explained 51.12% (Fig. 2) of the observed variation, with Axis 1 (35.9%) and Axis 2 (15.22%) (Fig. 2) capturing the majority of variance in genus-level community structure. Among the edaphic parameters analyzed, only soil Organic Matter (OM) content in the Caatinga biome exhibited a statistically significant association with bacterial assemblage differentiation (*P* < 0.003, *F* = 5.6043; Fig. 2). This finding underscore OM as a pivotal determinant of microbiome structuring across both rhizosphere and adjacent soil compartments in the mentioned area. Intriguingly, such OM-dependent structuring lacked statistical significance in the Restinga environment (*P* > 0.05), where bacterial taxonomic assemblages appeared decoupled from OM gradients.

### Rhizospheric and adjacent soil bacterial communities profiling based on 16S rDNA amplicon sequencing

The DNA samples extracted from the rhizosphere of *C. procera* and the adjacent soil showed sufficient quantity and quality for building libraries and 16S rDNA sequencing with the Illumina MiSeq platform (Table S1). Following rigorous quality filtering, a total of 114,126 high-quality reads were retained across all samples, which were subsequently clustered into 373 operational taxonomic units (OTUs).

**Fig. 3:**
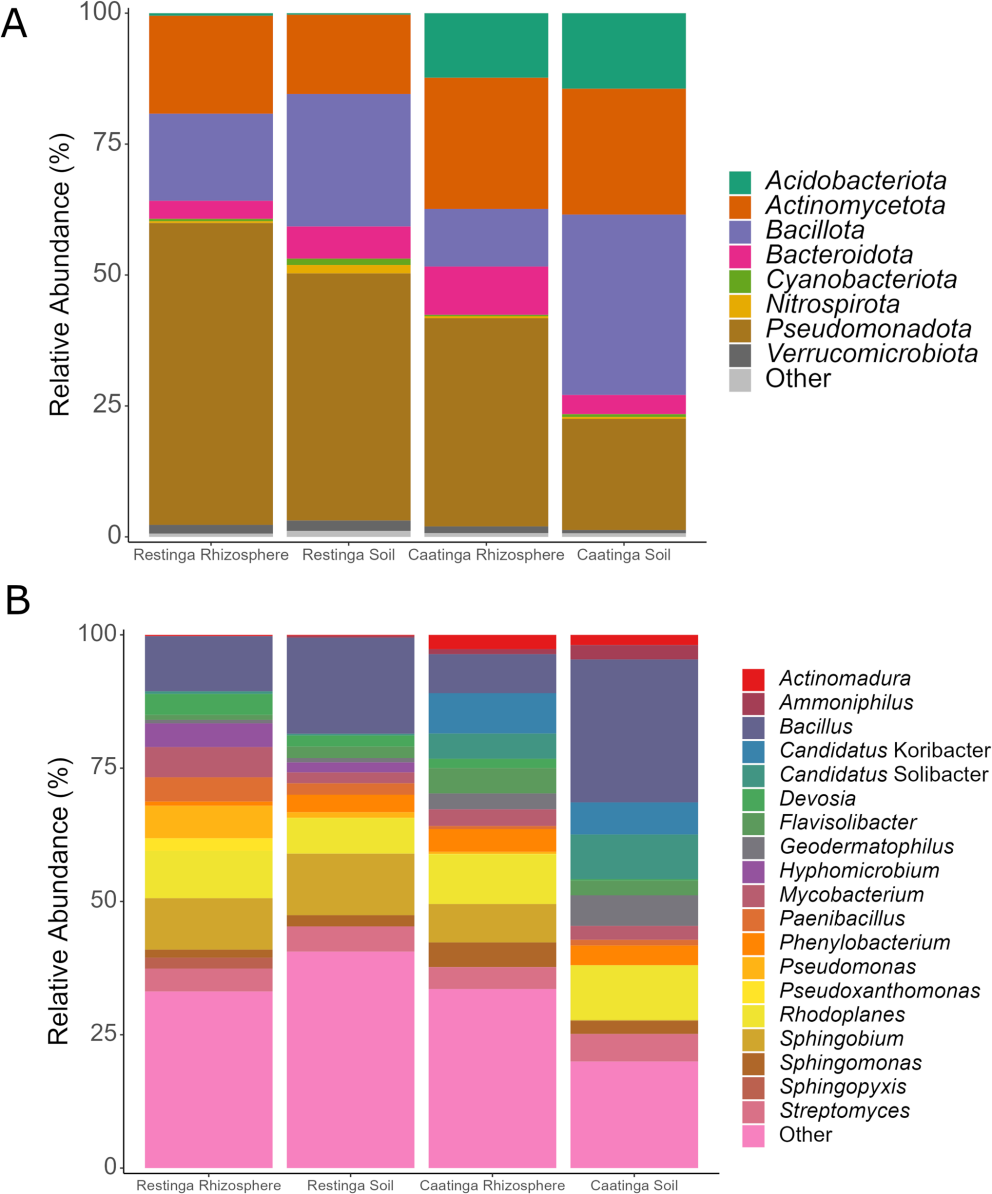
Taxonomic profiles (16S rDNA amplicon sequencing) of bacterial communities at the phylum **A** and genus **B** levels in the rhizosphere and adjacent soil from Restinga and Caatinga environments

Taxonomic classification revealed bacterial diversity spanning 21 phyla, 46 classes, 85 orders, 176 families, and 373 genera. The Pseudomonadota, Bacillota, Actinomycetota, and Acidobacteriota were the four most dominant phyla found in Restinga and Caatinga samples (Fig. 3A), both accounting for approximately 90% of bacterial OTUs. The remaining phyla with relative abundance < 1% were named as “others” (Fig. 3A).

Comparative analysis using the Kruskal–Wallis test demonstrated no statistically significant (P _BH_ > 0.05) disparities in the medians relative abundances of bacterial phyla across distinct soil niches (rhizosphere *vs.* adjacent soil) or environments (Restinga *vs.* Caatinga).

At the genus level, samples from the Restinga rhizosphere presented as the most abundant genera, respectively (Fig. 3B): *Bacillus*, *Sphingobium*, *Rhodoplanes*, *Pseudomonas*, *Mycobacterium*, *Paenibacillus*, *Hyphomicrobium*, and *Streptomyces*. In turn, the genera *Bacillus, Rhodoplanes, Streptomyces,* and *Phenylobacterium* were the most abundant in this environment's adjacent soil (Fig. 3B).

Considering Caatinga samples, the rhizosphere showed the following genera in decreasing order of abundance (Fig. 3B): *Rhodoplanes*, *Candidatus Koribacter*, *Bacillus*, *Sphingobium*, *Flavisolibacter,* and *Candidatus Solibacter*. The genera *Bacillus*, *Rhodoplanes*, *Candidatus Solibacter*, *Candidatus Koribacter*, *Geodermatophilus,* and *Streptomyces* were the most abundant in the Caatinga adjacent soil (Fig. 3B). As well as for the structure of the bacterial community at the phylum level, the medians obtained for the relative abundance at the genus level showed no significant difference (Kruskal–Wallis, P _BH_ > 0.05).

**Fig. 4:**
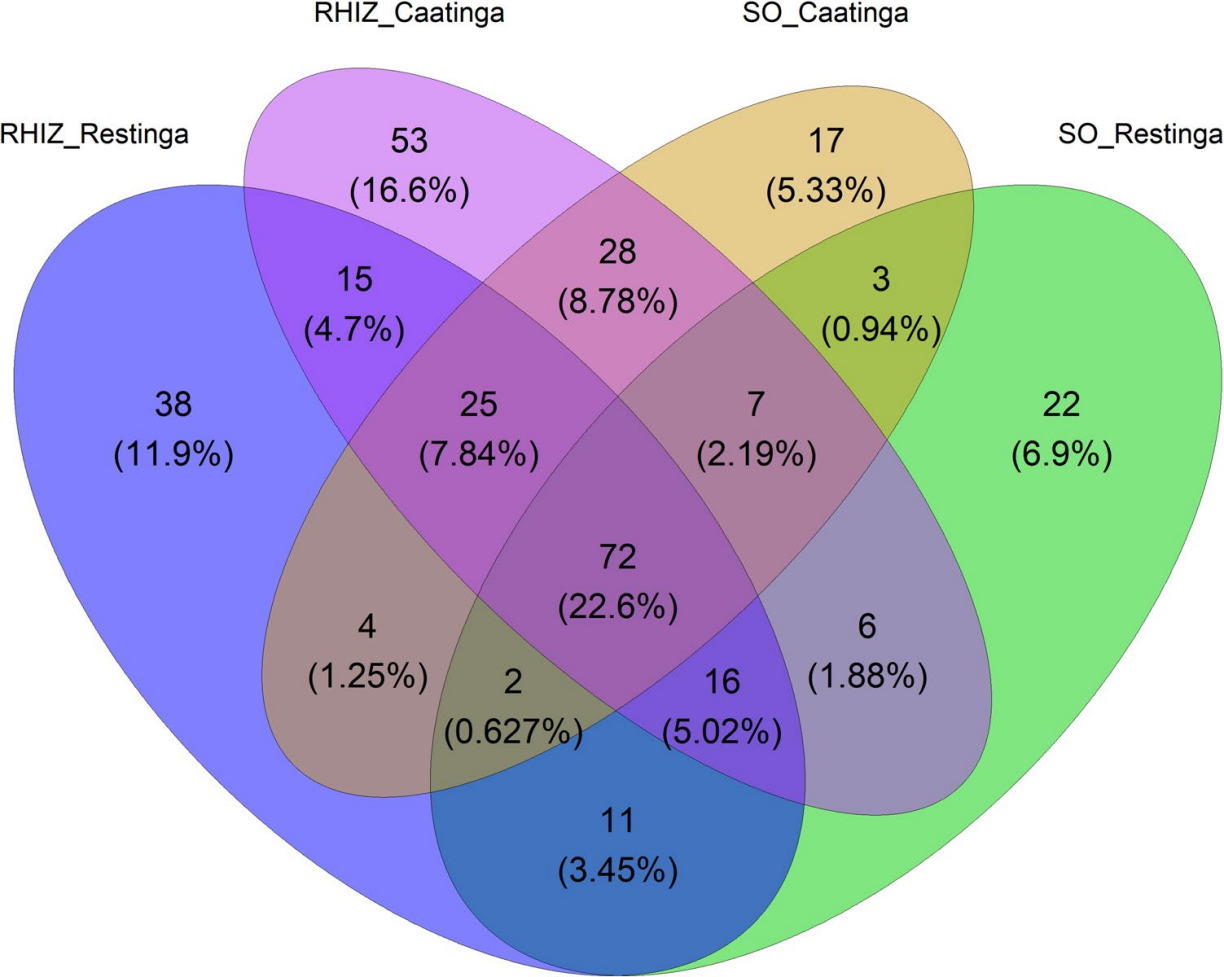
Venn diagrams representing the number of shared and exclusive OTUs among the two sample [rhizosphere (RHIZ) and adjacent soil (SO)] groups for both environments (Caatinga and Restinga)

From the total amount of identified OTUs, only 22.6% were shared between the *C. procera* rhizosphere and adjacent soil in both environments (Fig. 4A). Niche-specific OTUs revealed small disparities: 11.9% and 16.6% were exclusively associated with the Restinga rhizosphere and Caatinga rhizosphere, respectively. In comparison, 5.33% and 6.90% of the OTUs represented unique signatures of the Caatinga and Restinga adjacent soils, respectively (Fig. 4A). A total of 40.16% of the OTUs were shared between the rhizospheres of *C. procera* across the two biomes, whereas only 26.35% were shared between the respective adjacent soils. Within-biome comparisons revealed greater overlap between rhizosphere and adjacent soil in the Caatinga, with 41.41% of OTUs shared, compared to 31.70% in the Restinga (Fig. 4B, C).

### Diversity analysis of rhizospheric and adjacent soil bacterial communities

Alpha diversity assessments revealed no statistically significant disparities (Kruskal–Wallis, *P* > 0.05) between rhizosphere and adjacent soil niches or across the Restinga and Caatinga environments for Chao1 richness and Pielou’s evenness indices (Figure S2A, S2B). An interesting exception, however, emerged within the Caatinga ecosystem, where rhizosphere samples exhibited significantly higher bacterial abundance and community evenness, as quantified by the Shannon index (Kruskal–Wallis, *P* < 0.05; Dunn’s test, *P* = 0.0279; Figure S2C).

**Fig. 5:**
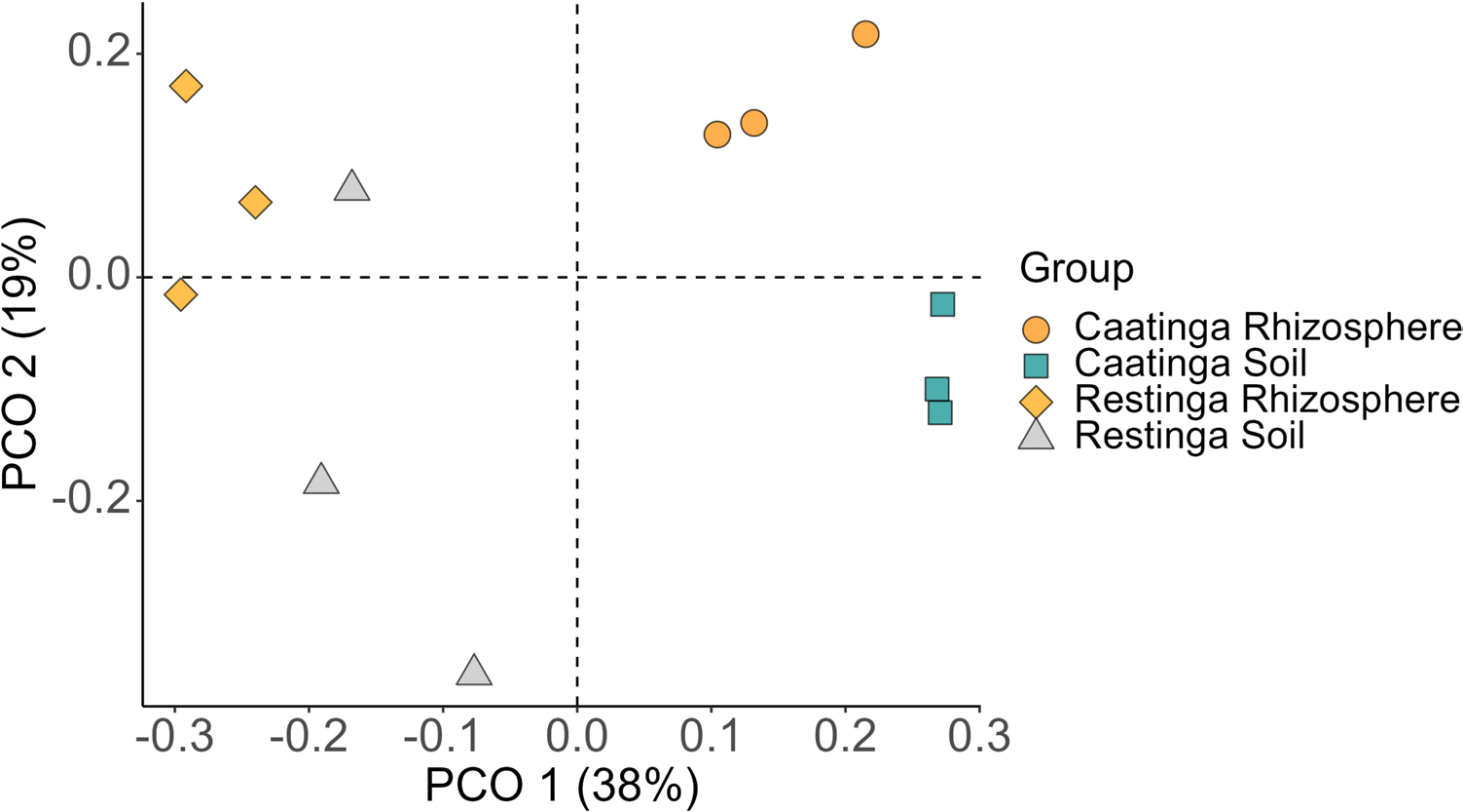
Bacterial community PCoA based on the Bray–Curtis distance between soil and rhizosphere samples of *C. procera* for each environment

Beta diversity analysis, employing principal coordinate analysis (PCoA) and PERMANOVA, demonstrated significant compositional divergence between Restinga and Caatinga communities (Fig. 5, Table S2). However, the test for homogeneity of multivariate dispersions of the group (betadisper) resulted in a significant value (permutest, *F* value = 6.6445, *p* = 0.03), indicating that the differences identified in community composition between environments can be partially attributed to artifacts of heterogeneous dispersion of groups. Furthermore, neither niche (rhizosphere *vs.* adjacent soil) nor the interaction between environments and niche exerted a statistically significant influence on community structure (Table S2).

### Analysis of bacterial networks

The network analysis revealed intricate topological architectures in the interactions among bacterial genera within the rhizosphere of the two studied ecosystems, with adjacent soil networks exhibiting markedly reduced connectivity (Table 2). Specifically, the rhizosphere networks of *C. procera* in the Restinga and Caatinga environments exhibited 57 and 60 nodes, respectively, interconnected by 74 and 71 edges. These networks demonstrated average node degrees of 2.59 and 2.36, alongside network diameters of 7 and 9 (Table 2). In stark contrast, the adjacent soil networks in these ecosystems displayed diminished complexity, with Restinga and Caatinga soils comprising only 26 and 18 nodes, 20 and 11 edges, diameters of 7 and 3, and average degrees of 1.53 and 1.22, respectively (Table 2).

**Table 2:** Topological properties of bacterial interaction networks in rhizosphere and adjacent soil from Restinga and Caatinga environments

Network metrics	Restinga		Caatinga	
	Rhizosphere	Soil	Rhizosphere	Soil
Number of nodes	57	26	60	18
Number of edges	74	20	71	11
Number of positive correlations	14 (19%)	2 (10%)	21 (30%)	2 (18%)
Number of negative correlations	60 (81%)	18 (90%)	50 (70%)	9 (82%)
Network diameter	7	7	9	3
Average degree	2.59	1.53	2.36	1.22

**Fig. 6:**
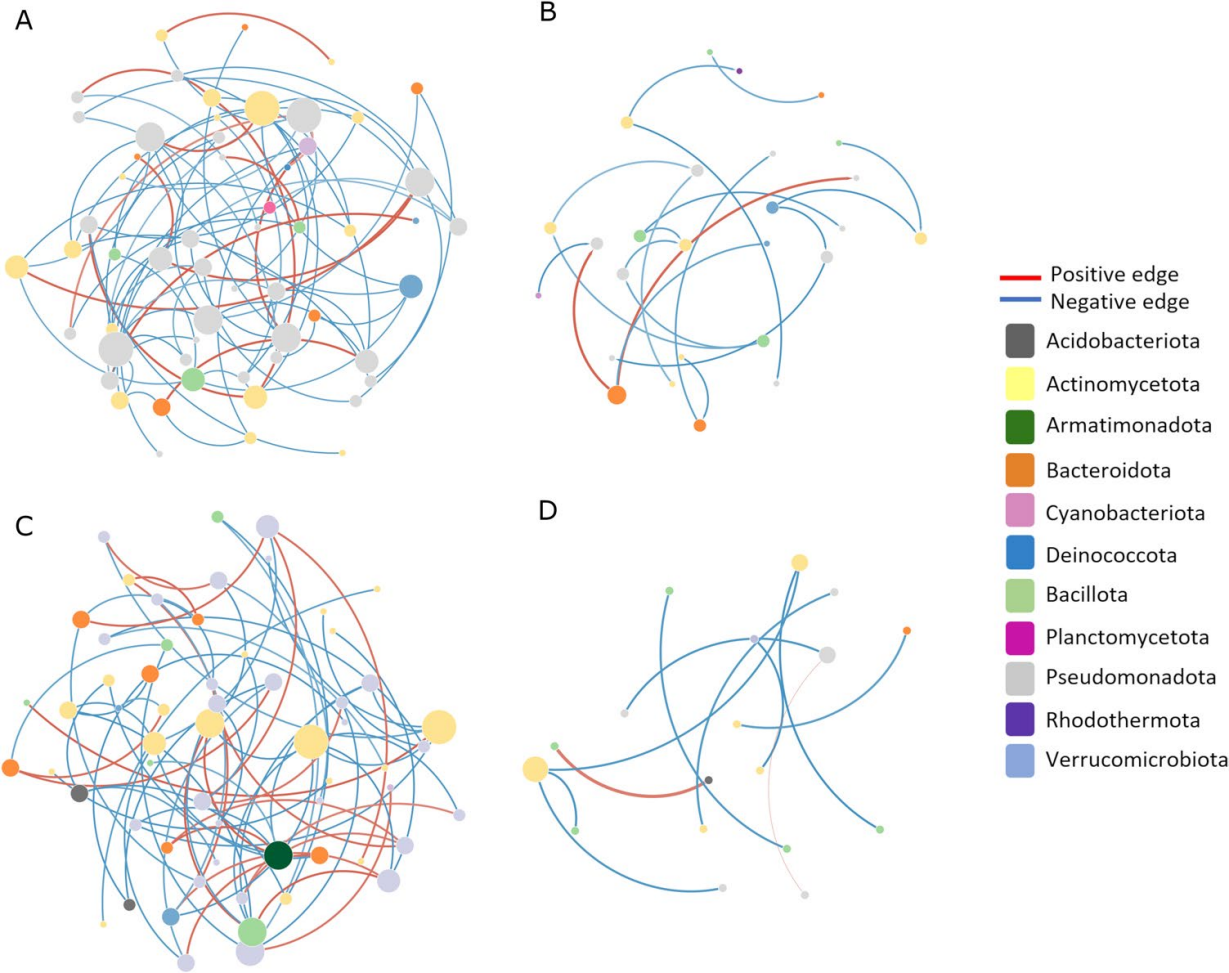
Networks of rhizosphere and soil bacterial communities in Restinga (rhizosphere – A and soil – B) and Caatinga (rhizosphere – C and soil – D) environments. Each node represents a different bacterial genus; the node size is proportional to the number of connections (i.e., degree). Each color represents the phyla to which the genera belong. Edge colors represent positive (red) and negative (blue) associations between two nodes. The wider the edge, the greater the degree of correlation

Despite the predominance of negative correlations across all networks (Fig. 6), rhizosphere-associated networks harbored a higher proportion of positive interactions compared to their adjacent soil counterparts in both environments (Table 2). Furthermore, a dominance of Pseudomonadota and Actinomycetota was observed across all networks, with these phyla collectively constituting most nodes, irrespective of habitat (Fig. 6).

### Analysis of the shotgun metagenomic sequencing dataset

DNA samples obtained from *C. procera* root endosphere showed adequate purity and concentration for library generation and sequencing on the Illumina HiSeq 2500 platform (2 × 150 bp) (Table S3). After quality filtering, approximately 301 million sequences (301,034,582) were obtained, with an average of 50 million sequences (min = 36,977,361, max = 58,410,795) per library for the taxonomic profile (Table S3).

### Profile of *C. procera* root endophytic microbial community based on shotgun sequencing

High-resolution shotgun metagenomic sequencing of the root endophytic microbiome of *C. procera* unveiled a taxonomically diverse consortium, encompassing 72 bacterial phyla (1108 genera; 98.02% of classified reads), 2 fungal phyla (51 genera; 1.28%), and 13 archaeal phyla (41 genera; 0.59%), across all root endosphere samples from the Caatinga and Restinga environments.

**Fig. 7:**
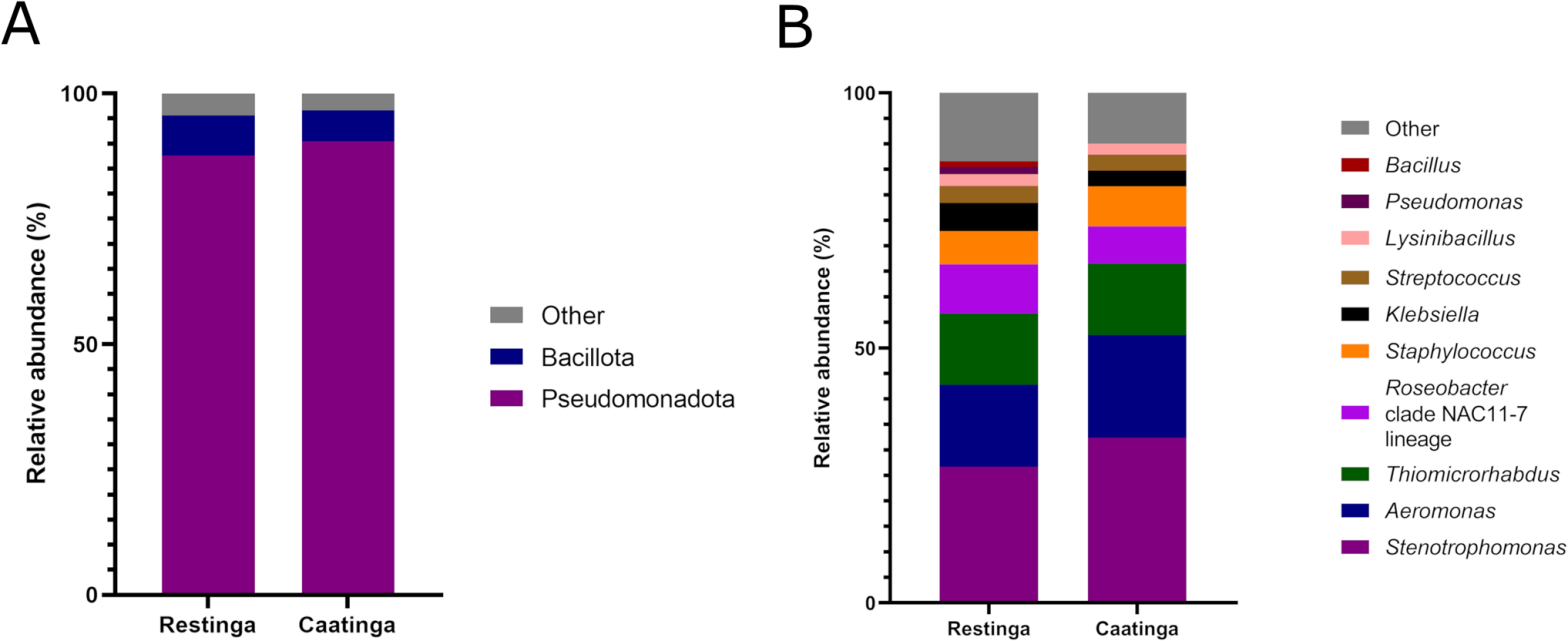
Taxonomic profile of *C. procera*'s bacterial communities of root endosphere in the Caatinga and Restinga environments at phylum **A** and genus **B** levels based on shotgun sequencing data

Within the bacterial domain, Pseudomonadota emerged as the dominant phylum, constituting 89% of the total bacterial community in both environments (Fig. 7A). Bacillota occupied a secondary position at 7%, while remaining phyla, collectively designated as “others”, exhibited relative abundances below 1% (Fig. 7A). Taxonomic stratification at the genus level revealed *Stenotrophomonas*, *Aeromonas*, *Thiomicrorhabdus*, the Roseobacter clade NAC11-7 lineage, and *Staphylococcus* as the most abundant taxa in both ecosystems (Fig. 7B). Subdominant genera exceeding 1% relative abundance included *Klebsiella*, *Streptococcus*, *Lysinibacillus*, *Bacillus*, and *Pseudomonas*, with residual taxa grouped under “others” (Fig. 7B).

**Fig. 8:**
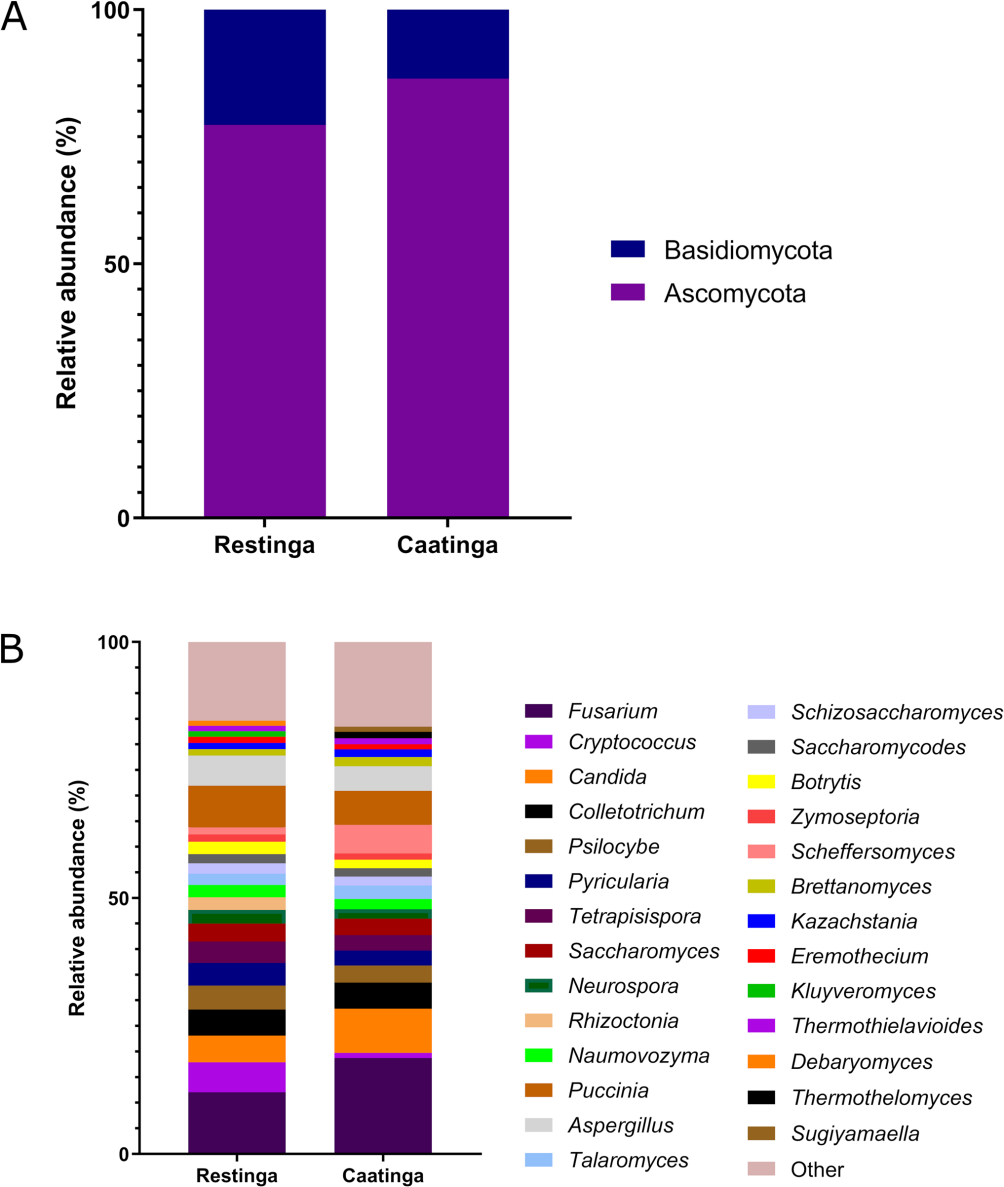
Taxonomic profile of root endosphere fungal communities of *C. procera* in the Caatinga and Restinga environments at phylum **A** and genus **B** levels based on shotgun sequencing data

Fungal phyla assemblages within the endosphere were dominated by Ascomycota and Basidiomycota, which together accounted for 81% of fungal sequences across both environments (Fig. 8A). At the genus level, *Fusarium* prevailed as the dominant taxon, with distinct subdominant hierarchies observed between environments: Restinga samples harbored *Puccinia*, *Aspergillus*, *Cryptococcus*, *Candida*, and *Colletotrichum;* whereas Caatinga samples exhibited a divergent profile dominated by *Candida*, *Puccinia*, *Scheffersomyces*, *Colletotrichum*, and *Aspergillus* (Fig. 8B).

**Fig. 9:**
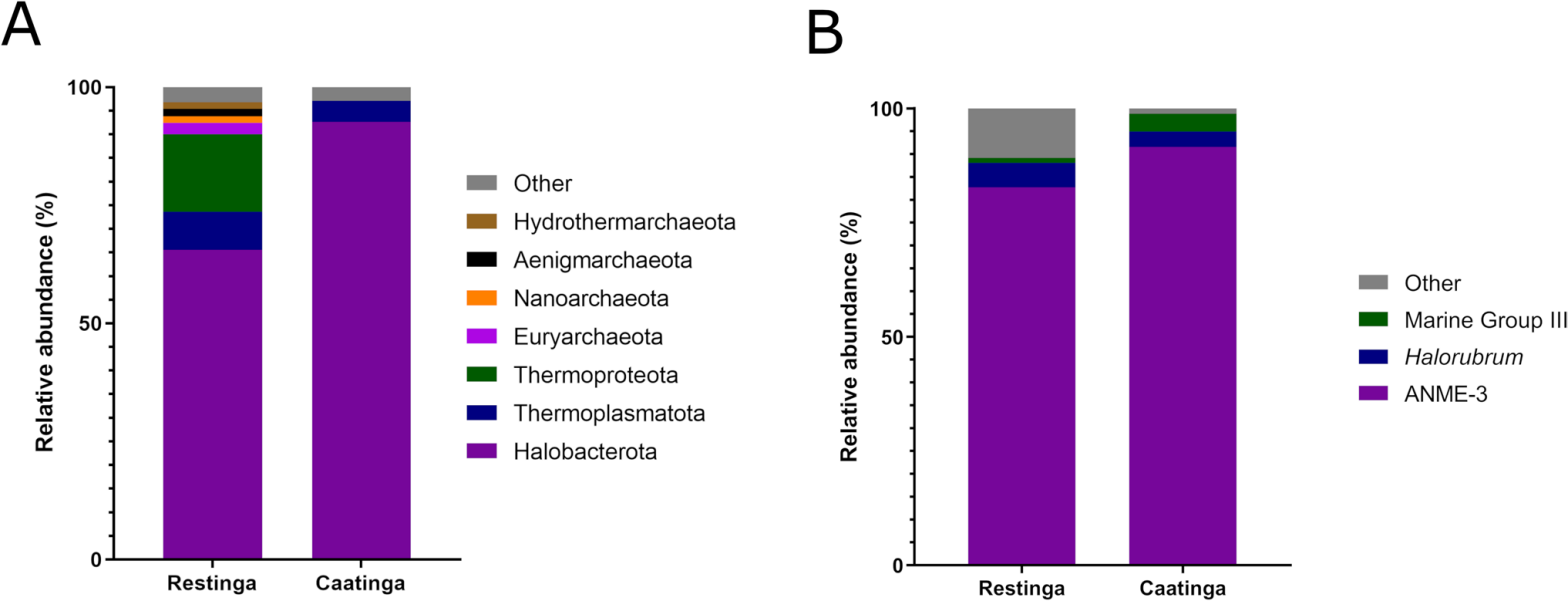
Taxonomic profile of root endosphere archaea communities of *C. procera* in the Caatinga and Restinga environments at phylum **A** and genus **B** levels based on shotgun sequencing data

Archaeal communities displayed striking environmental specificity. In Restinga, the phyla Halobacterota, Thermoproteota, and Thermoplasmatota co-dominated the endosphere, while Caatinga archaea exhibited a marked predominance of Halobacterota (92% relative abundance; Fig. 9A). Genus-level analysis identified ANME-3 as a ubiquitous dominant element, flanked by *Halorubrum* and Marine Group III in both environments (Fig. 9B).

### Alpha and beta diversity of the root endophytic microbiome

Alpha diversity metrics, including the Chao1, Shannon, and Pielou’s evenness indices, revealed no statistically significant differences (*P* > 0.05) in taxonomic richness, diversity, or evenness between bacterial and fungal communities inhabiting the root endosphere of *C. procera* across the Restinga and Caatinga environments (Figures S3A–S3C and S4A–S4C). In contrast, archaeal communities from the Restinga root endosphere exhibited significantly higher diversity and evenness, as indicated by the Shannon and Pielou’s indices, when compared to their Caatinga counterparts (Figure S5A–S5C), highlighting environment-specific archaeal niche partitioning.

**Fig. 10:**
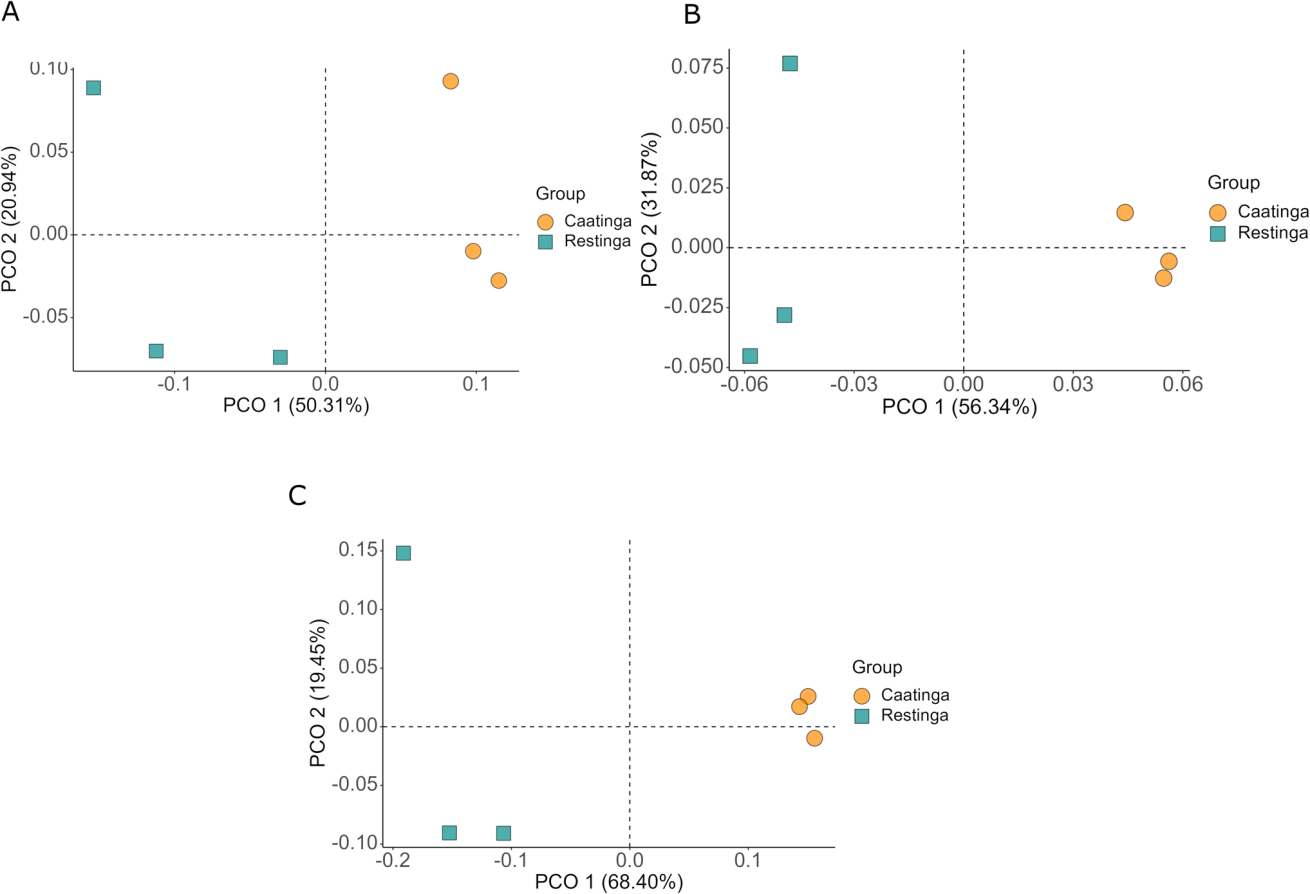
Principal coordinate analysis (PCoA) of bacterial **A**, fungal **B**, and archaeal **C** communities’ structure between samples (beta diversity) based on the Bray–Curtis distance

Beta diversity analysis, visualized through principal coordinate analysis (PCoA) of Bray–Curtis dissimilarities, demonstrated a clear clustering pattern separating endophytic microbial communities according to environment (Fig. 10). However, despite this spatial distinction, PERMANOVA results revealed no statistically significant compositional differences between Caatinga and Restinga samples for bacterial (*R*^2^ = 0.46046, *P* > 0.1), fungal (*R*^2^ = 0.50685, *P* > 0.1), and archaeal (*R*^2^ = 0.67027, *P* > 0.1) communities.

### Reassessing a priori hypotheses through the empirical evidence

At the inception of this investigation, three foundational hypotheses were formulated to unravel the mechanisms governing the microbial associations of *C. procera* (Box 1). These postulates were rigorously tested considering the experimental data, with their validity evaluated against statistically supported quantitative and qualitative findings.

**Box 1** Overview of the three hypotheses proposed in this study, along with their respective validation or rejection based on statistically supported results


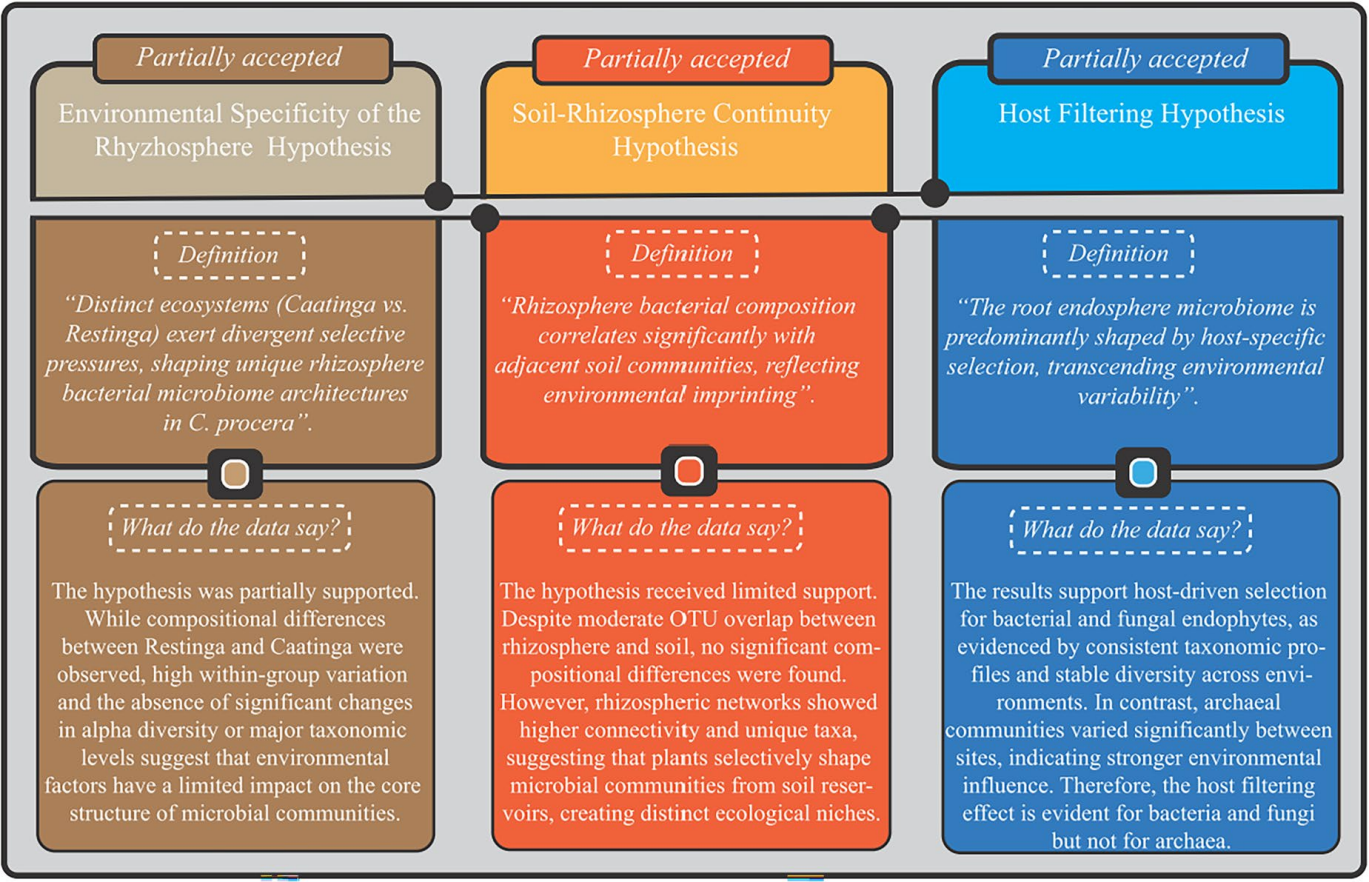


The environmental specificity of the rhizosphere hypothesis (Box 1) was partially supported. Beta diversity analyses revealed significant compositional divergence between Restinga and Caatinga rhizospheric bacterial communities, suggesting that environmental factors contribute to shaping community structure. However, this pattern was confounded by significant heterogeneity in group dispersions, and no significant differences were detected in alpha diversity or relative taxonomic abundance at the phylum or genus levels. Therefore, while environmental conditions appear to exert some influence on microbial community composition, the overall taxonomic architecture remains largely conserved, indicating that environmental pressure alone does not fully explain rhizospheric microbial assembly. Moreover, the interpretation of these patterns should be restricted to the scale of the sampled sites. Although the sites were selected within environmentally homogeneous areas in each ecosystem, the observed divergence should be regarded as contrasts between the specific locations investigated rather than as patterns necessarily broadly applicable to the entire ecosystem. Future studies incorporating multiple independent sites per ecosystem will be required to more robustly assess the influence of environmental pressures at a broader, regional scale.

The soil-rhizosphere continuity hypothesis received limited support from our findings (Box 1). While moderate OTU overlap occurred between rhizosphere and adjacent soil (41.4% in Caatinga vs. 31.7% in Restinga), analyses revealed: no significant niche differentiation in community composition (PERMANOVA, *P* > 0.05); and divergent ecological structures, with rhizosphere networks exhibiting 2.1–3.3 × greater node connectivity than adjacent soil (Table 2). These patterns suggest that adjacent soil serves as a microbial source pool, while plant-mediated selection preferentially enriches specific taxa (with 11.9% exclusive to the Restinga rhizosphere and 16.6% to the Caatinga rhizosphere) and reshapes microbial interactions, as reflected in higher positive edge-to-node ratios in rhizosphere networks. Thus, while environmental transmission can contribute to initial community assembly, the rhizosphere emerges as a plant-curated microhabitat with distinct ecological properties.

The host filtering hypothesis (Box 1) was supported for bacterial and fungal communities, but not for archaeal communities. Shotgun metagenomic analyses of the root endosphere revealed high taxonomic consistency across both environments, with Pseudomonadota, Ascomycota, and Basidiomycota dominating the bacterial and fungal compartments. Genus-level compositions were also highly conserved, and alpha diversity metrics showed no significant variation between Restinga and Caatinga. These findings support the idea that *C. procera* exerts strong host-driven selection on its bacterial and fungal endophytes, overriding environmental variability. In contrast, archaeal communities displayed significant differences in diversity and evenness between environments, suggesting that their assembly is more strongly influenced by abiotic environmental factors. Thus, the host filtering hypothesis is validated for dominant bacterial and fungal taxa, but its applicability to archaeal communities appears limited.

## Discussion

### Soil properties and diversity patterns of the rhizospheric bacterial community

The symbiotic interaction between *C. procera* and its microbiome remains sparsely documented, with current knowledge confined to characterizations of rhizospheric bacterial communities in Asian genotypes of this species (Al-Quwaie 2020; Ramadan et al. 2021). To address this critical knowledge gap and elucidate ecossystem-specific microbial assembly patterns, we conducted an integrative taxonomic profiling of rhizospheric, adjacent soil, and root endophytic microbiomes across two highly contrasting environments – the coastal Restinga and semi-arid Caatinga – of northeastern Brazil.

Edaphic analyses revealed marked divergence in soil physicochemical parameters between these regions. Notably, Caatinga soils exhibited significantly lower pH values compared to Restinga, though both environments fell within the acidic range (Table 1). Restinga soils demonstrated elevated phosphorus (P) and sodium ion (Na⁺) concentrations, whereas organic carbon (OC) and organic matter (OM) content were substantially enriched in Caatinga substrates, compared to Restinga soil. These findings align with prior edaphic surveys of these ecosystems (Rivas et al. 2020; Vieira et al. 2021), underscoring the distinct nutrient regimes inherent to each habitat.

Among the studied chemical properties, only OM significantly influenced the general structure of bacterial communities in the rhizosphere and adjacent soil to *C. procera* in the Caatinga environment. This data is consistent with previous studies that indicated OM among the main factors driving the structure of microbial communities in soil and rhizosphere (Guo et al. 2018; Ji et al. 2021). Soil OM is an important source of energy for the soil microbial community, as well as a source of essential nutrients for plants (Menšík et al. 2019). Intriguingly, the mentioned OM-dependent structuring was not statistically significant in the Restinga environment. This suggests ecosystem-specific divergences in the environmental filters influencing microbial community dynamics.

Despite the observed environmental divergences, our findings indicate that edaphic conditions are not the sole determinants of the rhizospheric microbiome composition in *C. procera*. Although beta diversity metrics revealed significant compositional dissimilarities between the bacterial communities inhabiting the rhizosphere in the Restinga and Caatinga biomes, this pattern was confounded by high within-group variability. Moreover, no statistically significant differences were observed in alpha diversity indices or in the relative abundance of dominant taxa at either the phylum or genus level. These results, when interpreted in conjunction with microbial co-occurrence network analyses (presented below), lend only partial support to our initial hypothesis (Box 1). While the contrasting edaphoclimatic conditions of Caatinga and Restinga do contribute to the differentiation of rhizospheric communities, the absence of marked taxonomic shifts at higher ranks suggests that such divergence is not predominantly compositional. Rather, it appears to be governed by ecological interactions and functional dynamics within the microbial consortia. Notably, the pronounced influence of organic matter (OM) in the Caatinga rhizosphere may have played a relevant role in the differentiation observed in beta diversity. As root exudates constitute a key component of rhizospheric OM, they can modulate nutrient availability and selectively shape microbial assembly (Grayston et al. 1997; Zhang et al. 2019). This reinforces the notion that plant-derived inputs, mediated by environmental context, may exert a more nuanced and functional form of selection, subtly sculpting the microbial landscape without inducing drastic changes in overall taxonomic profiles. That suggestion aligns with patterns observed in *Citrus* spp., where host-directed taxonomic selection was suggested to influence spatial heterogeneity in shaping rhizobacterial consortia (Xu et al. 2018). Similarly, *Phoenix dactylifera* L. (Asteraceae) sampled in different oases of the Sahara desert in Tunisia invariably selected similar rhizospheric bacterial communities, being responsible for the assembly of the microbiome in root niches (Mosqueira et al. 2019).

In other contexts, the negligible differences in beta diversity and the conserved alpha diversity metrics between rhizospheric and adjacent bulk soils in both biomes reinforce the paradigm that soil also acts as an edaphic reservoir for microbial recruitment (Mendes et al. 2014). This pattern is also evident in the rhizosphere of Dioon (Zamiaceae, Cycadales), where soil-derived taxa influence the microbiome composition (Suárez-Moo et al. 2019). In summary, a relationship between the rhizosphere and adjacent soil was confirmed, aligning with our second hypothesis. However, the rhizosphere is not merely a passive reflection of the surrounding soil – it actively selects and structures a distinct microbial community, marked by higher diversity and enriched microbial interactions. Thus, our second hypothesis was only partially supported (Box 1).

The results aforementioned collectively illuminate the dual role of *C. procera* as both a selective filter and a beneficiary of soil-borne microbial diversity, reconciling host specificity with environmental contingency in microbiome ecology.

### Composition of the rhizospheric bacteriome

At the phylum level, the rhizobacterial community of *C. procera* exhibited a conserved dominance profile, with Pseudomonadota, Bacillota, Actinomycetota, and Acidobacteriota collectively constituting the core microbiota – a pattern mirrored in rhizosphere analyses of conspecific populations from eastern Jeddah, Saudi Arabia (Al-Quwaie 2020; Ramadan et al. 2021). Taxonomic stratification at the genus level revealed a hierarchical abundance profile dominated by *Bacillus*, *Sphingobium*, *Rhodoplanes*, *Mycobacterium*, *Paenibacillus*, *Streptomyces*, *Candidatus Koribacter*, *Candidatus*, *Solibacter*, and *Flavisolibacter*. Notably, the consistent identification of *Bacillus* and *Streptomyces*, and *Mycobacterium* as keystone genera mirrors previous findings in *C. procera* rhizosphere (Ramadan et al. 2021), suggesting a conserved, host-specific mechanism of microbiome assembly, which may be partly driven by root exudates acting as selective chemical cues that filter and stabilize the microbial community across distinct environments. Plants secrete a wide range of root exudates (such as sugars, organic acids, amino acids, and flavonoids) that act as nutrient sources and signaling molecules, selectively enriching microbial taxa capable of utilizing or responding to them (Yang et al. 2025; Afzal et al. 2025). This chemically mediated filtering may contribute to the conserved dominance of specific phyla and keystone genera in *C. procera* rhizosphere.

In contrast to the present study conducted in environments invaded by *C. procera*, the genus *Ammoniphilus* was identified as the most abundant in its natural habitat's rhizosphere (Ramadan et al. 2021). This dichotomy underscores the ecological plasticity of *C. procera*’s microbiome assembly, wherein invasion-altered edaphic conditions may suppress native symbionts like *Ammoniphilus* while favoring cosmopolitan taxa adapted to perturbed environments. Such shifts in microbial guilds could reflect adaptive trade-offs between host-mediated selection and biogeochemical constraints imposed by novel ecosystems.

Among the identified genera, *Bacillus* is widely reported as a common inhabitant of the plant rhizosphere across diverse taxa, including those from semi-arid regions. Several isolates exhibit promising plant growth-promoting (PGP) traits, such as phosphate solubilization, exopolysaccharide (EPS) production, and drought tolerance (Rashid et al. 2022; Shah et al. 2022). *Sphingobium*, *Mycobacterium*¸ *Paenibacillus*, *Streptomyces,* and *Flavisolibacter* are genera commonly associated with the rhizospheric soil of other plant species and also have beneficial effects for PGP, such as phosphate solubilization, IAA production, siderophores, EPS, and ACC deaminase (Boss et al. 2022), also under water deficit conditions (Karmakar et al. 2021). Furthermore, *Rhodoplanes*, *Candidatus Koribacter,* and *Candidatus Solibacter* were also reported as members of the rhizosphere of other plant species, being related to C and N cycles (Zhang et al. 2022). Thus, our results indicate that *C. procera* recruits and establishes an association with some bacterial taxa that positively affect (PGP activities) its rhizosphere. This adaptive symbiosis may further explain its invasive success in anthropogenically disturbed Brazilian ecosystems as reported by Gonçalves-Oliveira et al. (2022). In these environments, microbial-mediated ecological advantages facilitate rapid niche colonization and resource exploitation.

The complexity of rhizosphere bacterial networks in terms of nodes, edges, and average degree was higher than in the adjacent soil. This result is consistent with analyses observed in previous studies for other taxa (Lee et al. 2019; Ceja-Navarro et al. 2021). Possibly the greater complexity in the mentioned niche is due to the exudation of root metabolites in the rhizosphere, which can strengthen interactions between the bacterial community (Wang et al. 2022). Our results showed that the rhizosphere of *C. procera* establishes an association with a complex bacterial network that, in turn, can influence the settlement of *C. procera* in the invaded environments in South America.

Negative correlations were prevalent in all networks, with a higher proportion of negative associations between nodes from the adjacent soil networks of both environments. Rhizosphere-associated networks, in turn, harbored a higher proportion of positive interactions compared to their adjacent soil counterparts in both environments. Although these negative correlations are indicative of competition in the community (Faust and Raes 2012), some studies have shown that the frequency of such correlations in the network may indicate greater network stability under disturbances since the community can return to its equilibrium more quickly after a perturbation (Coyte et al. 2015; Yang et al. 2022). Thus, the prevalence of negative correlations may indicate stable networks under the harsh conditions of the two environments, especially for the plain soil that consists of a less controlled environment when compared to the soil close to the root that forms the rhizosphere.

The higher proportion of positive interactions observed in the bacterial networks of the *C. procera* rhizosphere – compared to those in adjacent soils – suggests that the rhizosphere represents a more cooperative and functionally integrated niche. This pattern likely reflects host-mediated selection aimed at promoting microbial synergies. Such positive interactions often denote relationships like commensalism, mutualism, or metabolic facilitation, which can enhance the ecological stability and resilience of microbial communities within this compartment (Faust and Raes 2012).

### Root endophytic microbiome: structure and composition

#### The bacterial communities

Bacteria emerged as the dominant domain within the root endosphere microbiome of *C. procera* across both studied biomes, underscoring their pivotal role in plant–microbe symbiosis. The dominant bacterial phyla, Pseudomonadota and Bacillota, were also reported to be abundant among the endophytic bacterial community of plant roots when subjected to semi-arid conditions (Zhou et al. 2020), saline stress (Luo et al. 2021), in addition to invasive plants (Hong et al. 2015; Cheng et al. 2019) and in the succulent species *Opuntia ficus-indica* (L.) Mill. (Cactaceae) (Karray et al. 2020). Most bacterial genera abundant in the root endosphere of *C. procera* have also been identified as endophytic inhabitants in other plant species, where they are thought to play beneficial roles in promoting plant growth and health. For instance, *Stenotrophomonas*¸ dominant genus in root endosphere samples of *C. procera* in both environments, is adaptable to adverse environments and capable of colonizing host plants, especially in root and stem vascular tissues, conferring positive effects on tolerance to different stresses and aiding plant growth (Ryan et al. 2009). Such effects were identified from endophytic *Stenotrophomonas* bacteria isolated from other plant species (Tiwari et al. 2016; Ulrich et al. 2021). Furthermore, endophytic bacterial genera *Aeromonas*, *Lysinibacillus*, *Staphylococcus*, *Bacillus*, *Pseudomonas*, and *Klebsiella –* isolated across diverse plant taxa – have been extensively documented as multifunctional PGP symbionts, harboring a robust metabolic arsenal critical for host fitness. These taxa exhibit a suite of synergistic PGP mechanisms, including nitrogen fixation, phosphate solubilization, siderophore biosynthesis, indole-3-acetic acid (IAA) production, and 1-aminocyclopropane-1-carboxylate (ACC) deaminase activity (Kukla et al. 2014; Shabanamol et al. 2018; Yang et al. 2020; Li et al. 2022), besides salt and water stress tolerance (Bokhari et al. 2019; Fan et al. 2020; Pang et al. 2020). Thus, the abundance of these genera in the root endosphere of *C. Procera* may have relevance in efficiently adapting this species to the adverse conditions of the coastal and semi-arid environments.

The bacterial genera *Thiomicrorhabdus* and *Roseobacter* clade NAC11-7 were also identified with surprising abundance in the *C. procera* endosphere. This is the first report of *Thiomicrorhabdus* as a plant endophytic. It has been generally isolated from hydrothermal vents and coastal sediments; members of this genus are able to fix carbon (Scott et al. 2019) and include sulfur-oxidizing species (Liu et al. 2021). As it is thermophilic (Barosa et al. 2023), we suggest that *Thiomicrorhabdus* may contribute to the establishment of *C. procera* under abiotic stress conditions in the studied environments. The *Roseobacter* clade, in turn, was described as endophytic only in wheat (*Triticum aestivum* L., Poaceae) seeds (Aswini et al. 2023). The subgroup NAC11-7, specifically, identified abundantly in the roots of the evaluated organism, includes members associated with algae, algal blooms, and nearshore seawater (Buchan et al. 2005). This subgroup has not been reported to date as plant endophytes. Members of the *Roseobacter* clade possess diverse metabolic capabilities, including sulfur transformations and carbon monoxide oxidation (Buchan et al. 2005). In this context, we propose that, in addition to the already known symbiotic relationships of *Roseobacter* with several marine eukaryotic organisms (Buchan et al. 2005), the mentioned clade can establish symbiotic interactions with *C. procera*, which could be favorable for its survival under the conditions studied.

#### The fungal communities

The fungal endosphere community of *C. procera* comprised the phyla Ascomycota and Basidiomycota, which are also commonly found in the endospheric mycobiomes of other plant species from semi-arid regions (Coleman-Derr et al. 2016) and coastal zones (Luo et al. 2021). Among the fungal genera, *Fusarium* was identified as dominant. Although this genus includes many phytopathogens in different plant hosts (Ma et al. 2013), studies report its presence as endophytic, describing *Fusarium* strains as having PGP activities (Bilal et al. 2018; García-Latorre et al. 2023) with potential for biological control (Gkikas et al. 2021). Similar to *Fusarium*, *Puccinia,* and *Colletotrichum* also comprise phytopathogens in different plant species (Dheepa et al. 2016; Hafez et al. 2017). However, some studies highlight *Puccinia* species as promising sources of biocontrol agents in different herbs (Hanley and Groves 2002; Fauzi 2009; Kurose et al. 2012), while some *Colletotrichum* members have been reported as endophytes with favorable activities for plant growth (Hiruma et al. 2016; Ye et al. 2020).

*Aspergillus* has also been reported as an endophytic fungus in various plant species, with some isolates exhibiting PGP potential (Faddetta et al. 2021), including under salt-stress conditions (Badawy et al. 2021). Among the other subdominant fungal genera identified, *Candida*, *Cryptococcus*, and *Scheffersomyces* were reported as endophytic in different plant species (Zhang and Yao 2015; Manzotti et al. 2020; Kalu et al. 2021; Solanki et al. 2021; Bora et al. 2025). This recurrent association of yeasts and dimorphic fungi with plant species underscores their ecological versatility as niche-generalist symbionts, suggesting untapped potential for harnessing their metabolic repertoires in phytobiome engineering.

#### The archaeal communities

In the archaeal community’s context – which represented the smallest fraction among the identified microbial domains – the Halobacterota was the predominant phylum. Halobacterota was recently described as endophytic in seeds of *Cistanche phelypaea* (Desj.) Fern.Casas & M Lainz (Orobanchaceae) from Iberian coastal salt marshes (Petrosyan et al. 2023). Furthermore, it was detected in the rhizosphere of halophytic plant species in a semi-arid saline environment in China (Qiu et al. 2022). The salt content in the soil of the Restinha and Caatinga environments was possibly favorable for the survival of this phylum and consequent establishment as an endophyte in *C. procera*. Thermoproteota, the second most abundant phylum in endosphere samples in the coastal environment, was also described as an abundant endophyte in roots of *Panicum virgatum* L. (Poaceae) (Singer et al. 2019) and leaves of *Olea europaea* L. (Oleaceae) (Müller et al. 2015). Thermoproteota have been linked to ammonia oxidation and nitrogen cycling (Offre et al. 2013), which may enhance nitrogen availability in nutrient-poor or saline soils, such as those typical of coastal ecosystems. Furthermore, recent studies indicate that archaea, including Thermoproteota, might participate in osmoprotection and oxidative stress mitigation, supporting plant resilience in harsh environments (Zhalnina et al. 2018). For Thermoplasmatota, the third most abundant phylum, there are no reports in the literature of its identification as an endophytic of plants. However, its presence was reported in the rhizosphere of *Elaeis guineensis* Jacq. (Arecaceae) (Ding et al. 2023). This phylum is known for its association with organic carbon metabolism (Wan et al. 2021).

At the genus level, ANME-3 and Halorubrum – both extremophilic archaea – were the most abundant taxa identified in the root endosphere of *C. procera* across both analyzed environments. Anaerobic methanotrophic archaea ANME-3 have not been previously described as associated with internal plant tissues. Members of ANME-3 are able to participate in the process of anaerobic methane oxidation (Niemann et al. 2006). For example, its abundance has been reported in sediments from an underwater mud volcano (Lösekann et al. 2007) and marine sediments (Pastor et al. 2017). The genus *Halorubrum*, in turn, was detected in the root endosphere and rhizosphere of the cactus species *O. ficus-indica* in an arid climate gradient of Tunisia (Karray et al. 2020). Furthermore, members of this genus are often described as abundantly distributed in saline and hypersaline environments (Makhdoumi-Kakhki et al. 2012; Bachran et al. 2019). In a recent study, it was demonstrated that a rhizospheric isolate of this genus presented a good estimate of a PGP property (zinc solubilization), expanding the perspectives of using halophytic archaea in mitigating salt stress in plants (Naitam et al. 2023). Therefore, we suggest that the presence of this genus in *C. procera* roots may have some relationship with the growth of this plant in saline soils, such as those from the Caatinga and Restinga.

Finally, Marine Group III, another prominent group identified, comprises archaea typically found throughout the water column (Haro-Moreno et al. 2017). To date, its presence has not been reported within internal plant tissues. Although archaea represented the least abundant component of the *C. procera* endophytic microbiome, the dominance of three genera suggests that the roots of this species selectively recruit specific archaeal taxa – or, alternatively, that only certain archaeal groups possess the necessary adaptations to colonize this niche.

### Diversity patterns of the root endophyte microbiome

Alpha diversity analyses revealed no significant differences in bacterial and fungal diversity between root endosphere samples from the Caatinga and Restinga environments. In contrast, significant differences in archaeal alpha diversity were detected, as evidenced by the Shannon and Pielou’s indices. Archaeal diversity was higher in the *C. procera* root endosphere in the Restinga environment, potentially reflecting greater evenness of archaeal taxa commonly associated with saline conditions and elevated temperatures.

Beta diversity analyses, in alignment with patterns observed for rhizospheric bacterial communities, demonstrated that root endophytic microbial communities of *C. procera* did not differ significantly between the two environments. This trend is consistent with findings in other plant species adapted to arid or nutrient-poor conditions, such as *Cycas panzhihuaensis* (a gymnosperm native to China) when grown in natural versus cultivated settings (Zheng and Gong 2019), and two Cactaceae species endemic to Mexico (*Myrtillocactus geometrizans* and *Opuntia robusta*) across distinct semi-arid regions (Fonseca-García et al. 2016).

Together, these aforementioned findings partially validate our third hypothesis (Box 1), supporting the notion that *C. procera* selectively shapes its endophytic microbiome, particularly regarding dominant taxa. However, environmental influence remains relevant for subdominant microbial groups – such as archaea in the present case. This duality between host filtering and environmental modulation aligns with the “core microbiome vs. flexible microbiome” model (Shade and Handelsman 2012), wherein the host maintains a conserved functional core while peripheral taxa vary according to external conditions.

The conservation of the called “core microbiome” raises two compelling possibilities: first, that *C. procera* may actively select from the environment dominant microbial taxa (bacteria and fungi) with which it maintains close mutualistic associations; and second, that the referred plant may vertically transmit a portion of its microbiome through seeds – an intriguing possibility that warrants further investigation in *C. procera* and has already been validated for other plant species, including cassava, soybean, pea, sugarcane, and coffee, among others (Johnston-Monje et al. 2021).

Contemporary research has progressively emphasized the potential inheritance of microbial communities across successive plant generations (Abdelfattah et al. 2023), indicating vertical persistence in plant–microbe holobionts driven by reciprocal evolutionary processes. The transfer of seed-dwelling microbiota to offspring holds increasing scientific relevance due to its role in modulating individual plant responses to localized environmental pressures (which governs survival outcomes and ecological performance) and owing to its contribution to deciphering the evolutionary interplay between plants and their symbiotic microbiomes (Cardinale and Schnell 2024). Evolutionarily, plants gain selective advantages by incorporating beneficial microbial consortia into their seeds and transferring these to descendants, thereby bolstering survival probabilities and adaptive resilience in future generations. Prolonged evolutionary cycles may solidify these synergistic associations as conserved attributes within holobiont systems (Rahman et al. 2018).

## Conclusion

The present study is the first to characterize the structure of the rhizospheric and endophytic microbiomes of *C. procera* populations outside of their native area (Africa and Asia). For this, we analyzed individuals from the Caatinga and Restinga environments of northeastern Brazil, using both shotgun metagenomics and 16S rDNA amplicon sequencing. It is important to note that the differences observed may reflect the specific locations analyzed rather than the ecosystems as a whole. More general conclusions will require studies that include multiple independent sites per ecosystem to evaluate regional environmental effects. The evaluated data indicate that environmental conditions partially shaped the taxonomic composition of both fungal and bacterial communities in the root endosphere and rhizosphere of *C. procera*. However, most of these communities were remarkably similar across the two sampling sites, despite their geographic separation and contrasting soil chemical properties. This indicates a host-mediated regulation of root microbiome composition in *C. procera*, whereby the plant may selectively recruit specific taxa from prevalent soil microbial communities (e.g., through root exudates) or vertically transmit a conserved subset of its microbiome via seeds. In contrast, archaeal diversity was higher in the root endosphere of *C. procera* in the Restinga environment, suggesting a greater sensitivity of subdominant microbial groups to environmental variation. This observed pattern supports the notion of a dual influence on microbiome assembly, in which host filtering predominantly shapes the “core microbial” community. At the same time, environmental factors modulate the composition of more flexible, peripheral taxa. Such dynamics are well described by the “core microbiome vs. flexible microbiome” model. Additionally, it was observed that the root endosphere includes some members never described as plant endophytes, some of them extremophilic. We found some genera (especially bacterial) in relatively high abundance in the two plant niches analyzed, reported as beneficial in promoting plant growth. Possibly, the identified groups play a relevant role in the adaptation and developmental success of the species in environments with challenging conditions. More complex bacterial interaction networks observed in the rhizosphere of *C. procera*, in contrast to the adjacent soil, indicate the influence of this niche on the response of the associated bacterial community. Together, our results contributed to broadening the understanding of the characterization of the microbiome associated with *C. procera* and provided relevant information about its interaction with diverse microbial communities that may be explored in the future for the isolation of candidates with biotechnological potential to improve agricultural productivity and sustainability in arid and saline environments.

## Supplementary Information

Below is the link to the electronic supplementary material.

Supplementary file1 (PDF 596 KB)

## Data Availability

The genome data are available in the NCBI repository under BioProject PRJNA1053370.
