# Supplementary material for: Unlocking the microbiome of an extremophile plant: metagenomic insights into Calotropis procera’s endo-rhizosphere communities
Source: World J Microbiol Biotechnol. 2026 Mar 24;42(4):157. doi: 10.1007/s11274-026-04902-4 (PMC13013160; doi:10.1007/s11274-026-04902-4)
Supplement: Supplementary file 1 — Supplementary file1 (PDF 596 KB) [file 11274_2026_4902_MOESM1_ESM.pdf]

SUPPLEMENTARY MATERIAL

Unlocking the Microbiome of an Extremophile Plant: Metagenomic Insights into *Calotropis procera*'s Endo-Rhizosphere Communities

Thamara de Medeiros Azevedo[1], Flávia Figueira Aburjaile[2], Valesca Pandolfi[1], José Ribamar Costa Ferreira Neto[1,3], Giselle Gomes Monteiro Fracetto[4], Roberta Lane de Oliveira Silva[1], Rodrigo César Gonçalves-Oliveira[5], Vasco Ariston de Carvalho Azevedo[6], Bertram Brenig[7], Ana Maria Benko-Iseppon[1]

[1]Universidade Federal de Pernambuco, Centro de Biociências, Departamento de Genética, Recife, PE, Brazil.

[2]Universidade Federal de Minas Gerais, Departamento de Medicina Veterinária Preventiva, Belo Horizonte, MG, Brazil.

[3]Embrapa Soja – Brazilian Agricultural Research Corporation (Embrapa), Distrito de Warta, Londrina, PR, Brazil.

[4]Universidade Federal Rural de Pernambuco, Laboratório de Microbiologia e Bioquímica do Solo, Recife, PE, Brazil.

[5]Universidade Federal de Pernambuco, Petrolina, PE, Brazil.

[6]Universidade Federal de Minas Gerais, Departamento de Genética, Ecologia e Evolução, Belo Horizonte, MG, Brazil.

[7]University Göttingen, Department of Molecular Biology of Livestock, Germany.

CORRESPONDING AUTHOR: Ana Maria Benko-Iseppon, Departamento de Genética, Centro de Biociências, Universidade Federal de Pernambuco, Av. Prof. Moraes Rego, 1235 – Cidade Universitária, Recife PE/CEP: 50670-901, Brazil. E-mail: ana.iseppon@ufpe.br

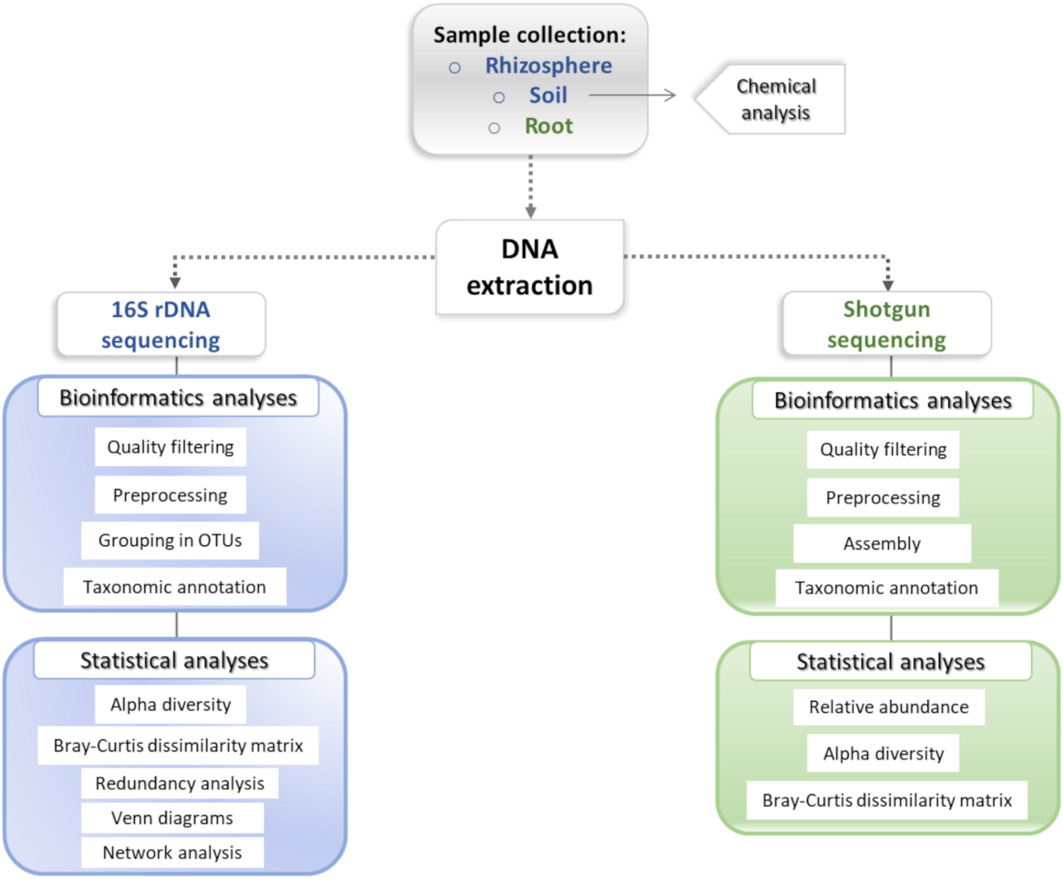

Figure S1. Workflow of analyses applied in the study. On the left side (in blue), analyses using rRNA coding sequences (rDNA) applied to the rhizosphere and soil. On the right side, the root endosphere analyses based on shotgun sequencing.

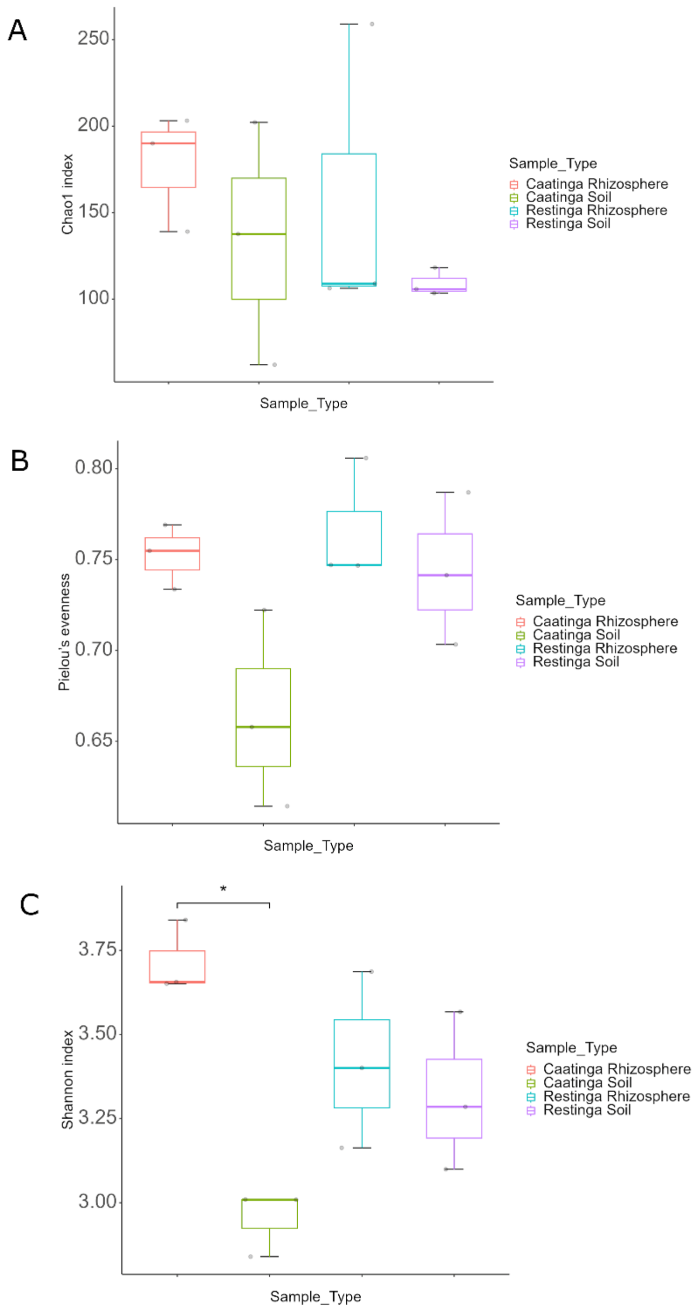

Figure S2. Boxplots of alpha diversity indices (Chao1 - A, Pielou's - B and Shannon - C) of bacterial communities in *C. procera* rhizosphere and adjacent soil samples in Caatinga and Restinga environments (* $P < 0.05$).

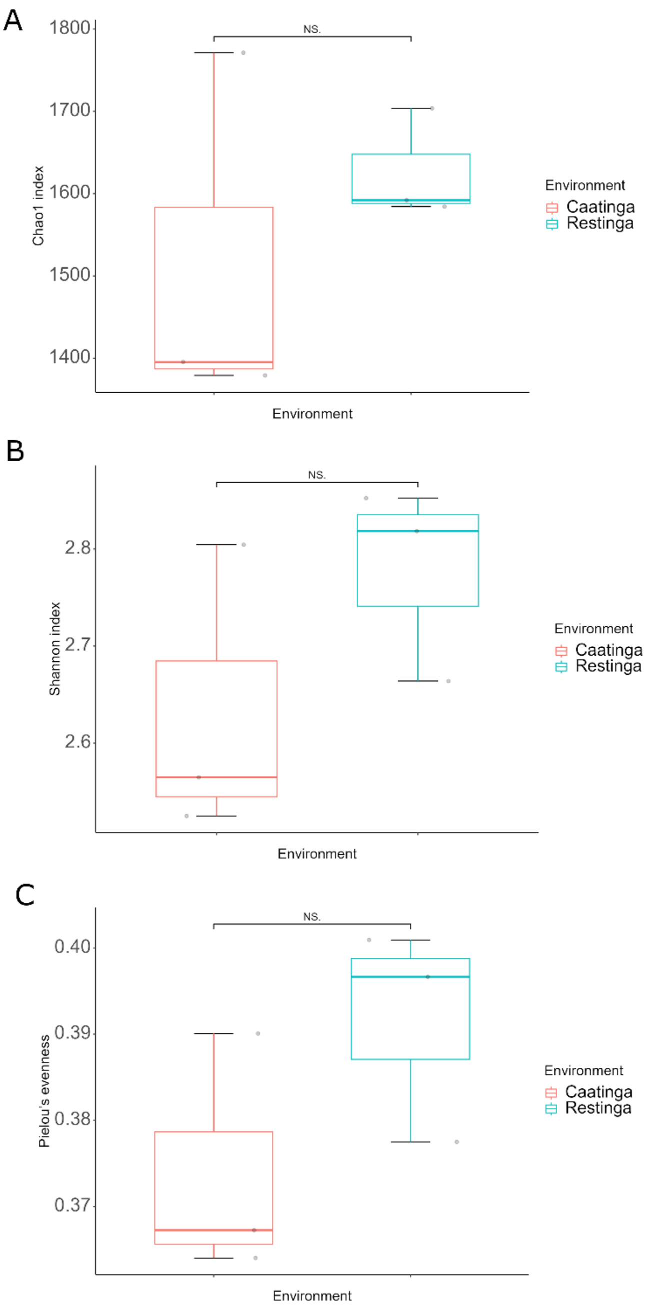

Figure S3. Boxplots of alpha diversity indices (Chao1 – A, Shannon – B, and Pielou's – C) of bacterial communities in root endosphere samples of *C. procera* in the Caatinga and Restinga environments. NS does not indicate statistical significance.

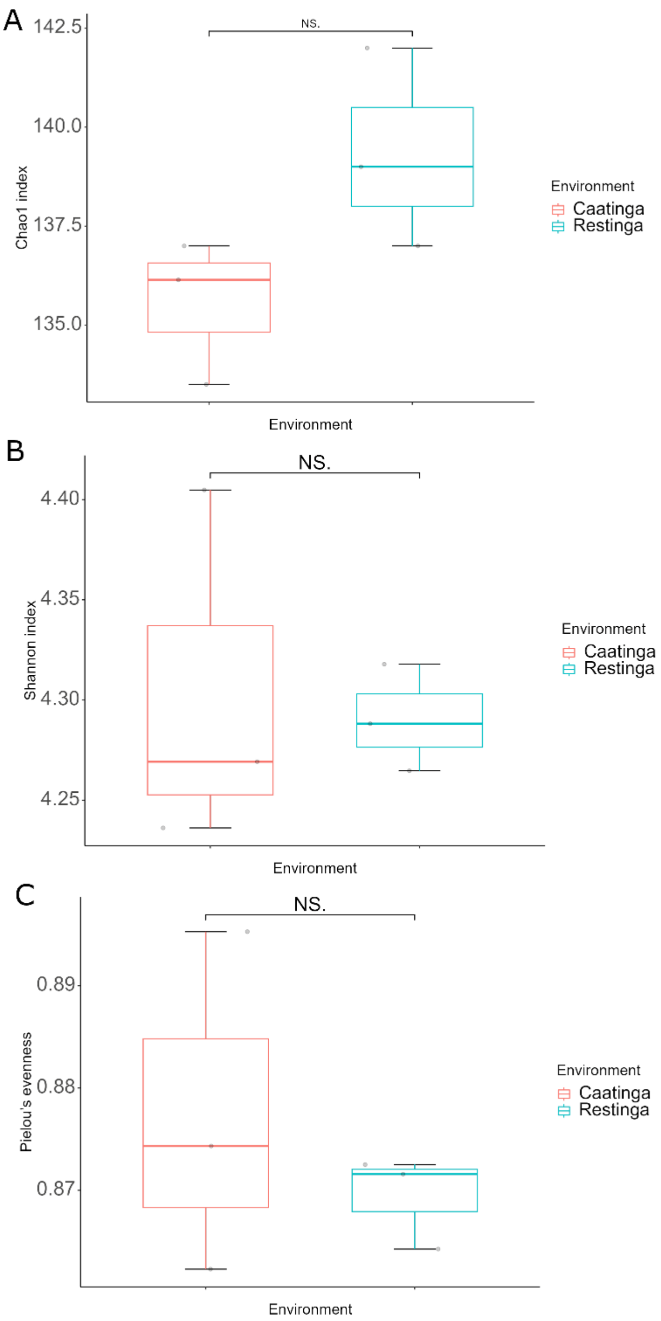

Figure S4. Boxplots of alpha diversity indices (Chao1 – A, Shannon – B and Pielou's – C) of fungal communities in root endosphere samples of *C. procera* in the Caatinga and Restinga environments. NS does not indicate statistical significance.

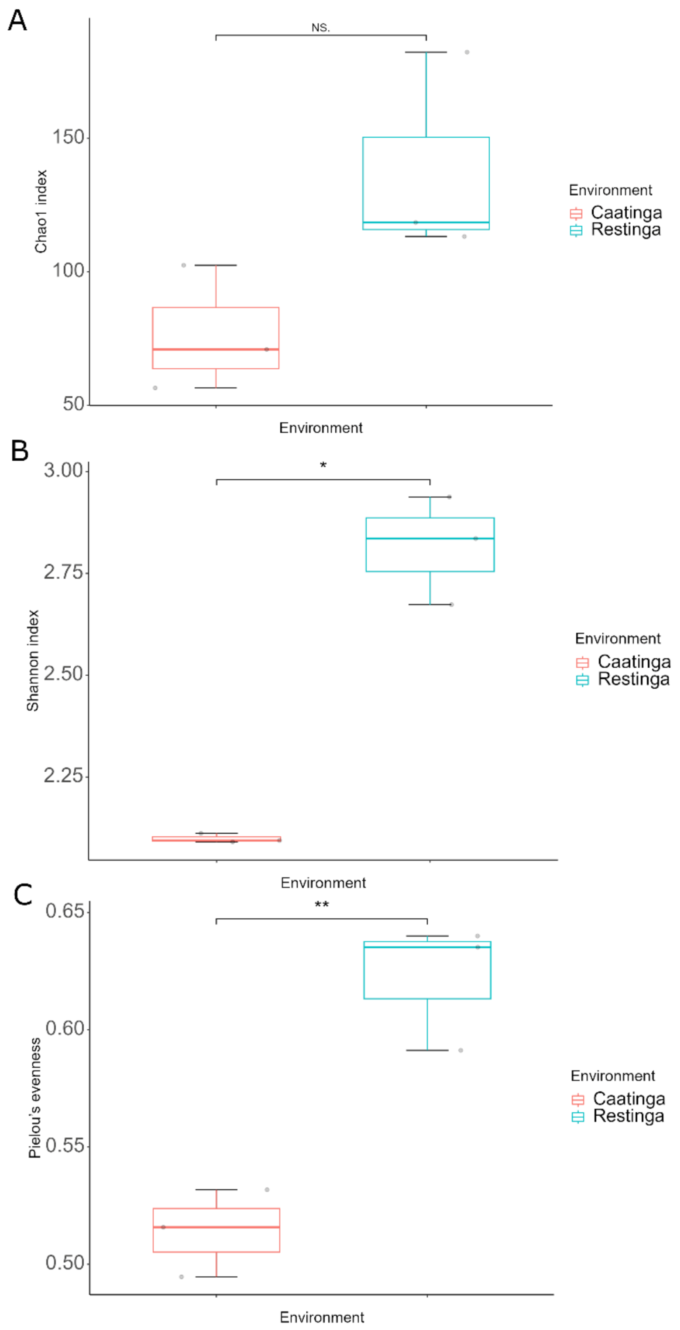

Figure S5. Boxplots of alpha diversity indices (Chao1 – A, Shannon – B and Pielou's – C) of archaeal communities in root endosphere samples of *C. procera* in the Caatinga and Restinga environments. Asterisks indicate statistically significant differences (* P < 0.05; ** P < 0.01) and NS does not indicate statistical significance.

Table S1. Major properties of extracted DNA after quality control

Sample	Extracted DNA (QC)			
	Concentration (ng/μl)	OD260/OD280	OD260/OD230	Amount (ng)
Rhiz1	1.5	1.83	0.54	100
Rhiz2	2.0	1.89	0.90	100
Rhiz3	3.0	1.90	0.70	100
So1	0.750	1.8	0.52	100
So2	0.578	1.8	0.50	100
So3	0.410	1.81	0.60	100
Rhiz4	5.10	2.06	0.65	100
Rhiz5	5.00	1.82	0.73	100
Rhiz6	4.8	2.03	0.66	100
So4	1.66	1.7	0.34	100
So5	1.8	1.81	0.44	100
So6	1.9	1.9	0.44	100

Rhiz and So 1, 2, and 3 are rhizosphere and soil samples of Restinga. Rhiz and So 4, 5, and 6 are rhizosphere and soil samples of Caatinga. OD: Optical Density measured by Nanodrop-2000. GC: Guanine + Cytosine.

Table S2. Results of PERMANOVA analysis

**Source of variation**	**Df**	**Sum of Sqs**	R^2	F	***p*-value**	**permutest**
**Environment**[a]	1	0.55387	0.35834	5.584595	0.004 **	0.03 *
**Compartiment**[b]	1	0.24612	0.15923	1.893924	0.08	0.808
**Environment x Compartment**	1	0.12461	0.08062	1.6052	0.124	
**Residuals**	8	0.62105	0.40180			
**Total**	11	1.54566	1.00000			

PERMANOVA was conducted with 999 permutations using data from OTUs. Df = degrees of freedom; Sum Of Sq = sum of squares; R^2 = coefficient of determination; F = pseudo-F ratio; p and permutest values, respectively, followed by asterisks indicate statistical significance (* $p < 0.05$, ** $p < 0.01$). [a] Two environments (Restinga and Caatinga), [b] Two sampled niches (soil and rhizosphere).

Table S3. Major properties of extracted DNA after quality control and results of sequencing with Illumina HiSeq2500

Sample	Extracted DNA (QC)				Libraries
	Concentration (ng/µl)	OD260/OD280	OD260/OD230	Amount (µg)	
Res1	11.1	1.86	2.01	1.14	Root R1
Res2	6.4	1.81	2.06	1.25	Root R2
Res3	17.7	1.84	2.07	1.41	Root R3
Caa1	6.53	1.89	2.02	1.17	Root S1
Caa2	9.8	1.85	2.08	1.12	Root S2
Caa4	9.24	1.83	2.01	1.18	Root S4

Libraries	Illumina HiSeq2500 sequencing	
	Total reads (QC)	%GC
Root R1	58410795	43
Root R2	58311351	33
Root R3	56638271	35
Root S1	43659468	35
Root S2	36977361	34
Root S4	47037336	41

Res 1, 2 and 3 are root endosphere of Restinga.
Caa 1, 2 and 3 are root endosphere of Caatinga.
Root R 1, 2, and 3: (root endosphere of Restinga DNA libraries): libraries made from 3 samples.
Root S 1, 2 and 4: (root endosphere of Caatinga DNA libraries): libraries made from 3 samples.
QC: Quality Control. OD: Optical Density measured by Nanodrop-2000. GC: Guanine + Cytosine.
